# Optical control of electrons in a Floquet topological insulator

**DOI:** 10.1038/s41567-026-03429-7

**Published:** 2026-08-31

**Authors:** Daniel M. B. Lesko, Tobias Weitz, Simon Wittigschlager, Weizhe Li, Christian Heide, Ofer Neufeld, Peter Hommelhoff

**Affiliations:** 1 Department of Physics, Friedrich-Alexander-Universität Erlangen-Nürnberg, Erlangen, Germany; 2 Fakultät für Physik, Ludwig-Maximilians-Universität, Munich, Germany; 3 Department of Physics, University of Central Florida, Orlando, FL USA; 4 CREOL, The College of Optics and Photonics, University of Central Florida, Orlando, FL USA; 5 Schulich Faculty of Chemistry, Technion - Israel Institute of Technology, Haifa, Israel; 6 Max Planck Institute for the Structure and Dynamics of Matter, Hamburg, Germany

**Keywords:** Topological insulators, Ultrafast photonics, Nonlinear optics

## Abstract

Light-dressed materials offer a route to generating electronic properties that differ from those of materials at equilibrium. The resulting band structures can host distinct quantum and topological phenomena. So far, optical control of charge within a light-dressed band structure has remained elusive. Here we demonstrate optical control of electrons in light-dressed graphene. By focusing circularly polarized femtosecond laser pulses at 1,550 nm on monolayer graphene, we generate a Floquet topological insulator. With a phase-locked second-harmonic field, we dynamically control electrons in this state, a technique we call harmonic Floquet spectroscopy. We observe photocurrent circular dichroism, an all-optical anomalous Hall effect and valley-polarized currents. The photocurrents show strong subcycle phase sensitivity, suggesting a route to ultrafast control in topological electronics, spectroscopy and attosecond physics in quantum materials.

## Abstract (web summary)

Driving a solid with light can create a topological insulator, but controlling its electrons has remained elusive. Optical control of electrons in a Floquet topological insulator has now been achieved in light-dressed graphene.

## Main

One interpretation for understanding the interaction of light with matter is through light-induced modification of the material’s electronic band structure. The oscillatory and periodic nature of the light field generates new light-dressed states, described by the Floquet theorem (the time analogue of the Bloch theorem), generating periodic bands spaced by the dressing energy. These new quasi-static light-dressed states can exhibit new quantum and topological properties, transformed from their original material eigenstates by the amplitude, phase and symmetry of the optical dressing field. A particularly exciting class of these light-dressed materials are Floquet topological insulators (FTIs), where a topologically trivial material is transformed into a topological insulator by light dressing. FTIs represent out-of-equilibrium systems showing intriguing topological properties not present in the undressed material. While first predicted for optically driven solids^1–4^, most insights into Floquet topological physics have been obtained with photonic waveguides and cold atoms in optical lattices^5–10^. Experiments in synthetic systems have shown the fascinating ability to design and control material properties with the Floquet Hamiltonian, elucidating both static and time-dependent phenomena^11,12^.

In solids, FTIs promise to offer high tunability with the driving laser field properties and the choice of the underlying material system. While theory suggests that Floquet dressing can synthesize almost arbitrary material band structures with varying topological phenomena^3,4,13^, the optical control of Floquet topological states in real materials remains an outstanding challenge. Pioneering works observed Floquet–Bloch bands on a topological surface state^14^, as well as engineered bands in a bulk semiconductor^15^. In a wide-bandgap semiconductor, ultrafast Floquet physics can be used to explain phenomena such as the enhancement of optical nonlinearities^16^. Importantly, a differential d.c. anomalous Hall conductivity was shown in ref. ^17^, acting as a clear indirect signature of Floquet topology.

Here, we demonstrate a new method for generating and probing Floquet topological phenomena by harmonically related optical fields (and without a d.c. field), which we call harmonic Floquet spectroscopy. We utilize graphene, a well-understood topologically trivial semi-metal, with an equilibrium band structure that lacks Berry curvature. We generate an FTI by light dressing graphene with circularly polarized femtosecond laser pulses, and subsequently control and drive electrons within this state using a second-harmonic optical field, generating photocurrents. Intriguingly, strongly dressing the band structure opens topological bandgaps at harmonics of the dressing energy, allowing us to probe topological physics in a unique way^18^, and in a vastly different region of the dressed band structure than explored previously^17^. We measure the all-optical anomalous Hall effect, photocurrent circular dichroism and a Berry curvature photocurrent enhancement. Our novel approach enables control and simultaneous analysis of topological and sub-optical-cycle (attosecond) phenomena, combining the traditionally static picture of a dressed band structure with the sub-optical-cycle micromotion of the FTI state. Here we accomplish this and pave the way to Floquet topotronics and its all-optical implementation.

## FTI state generation

**Fig. 1:**
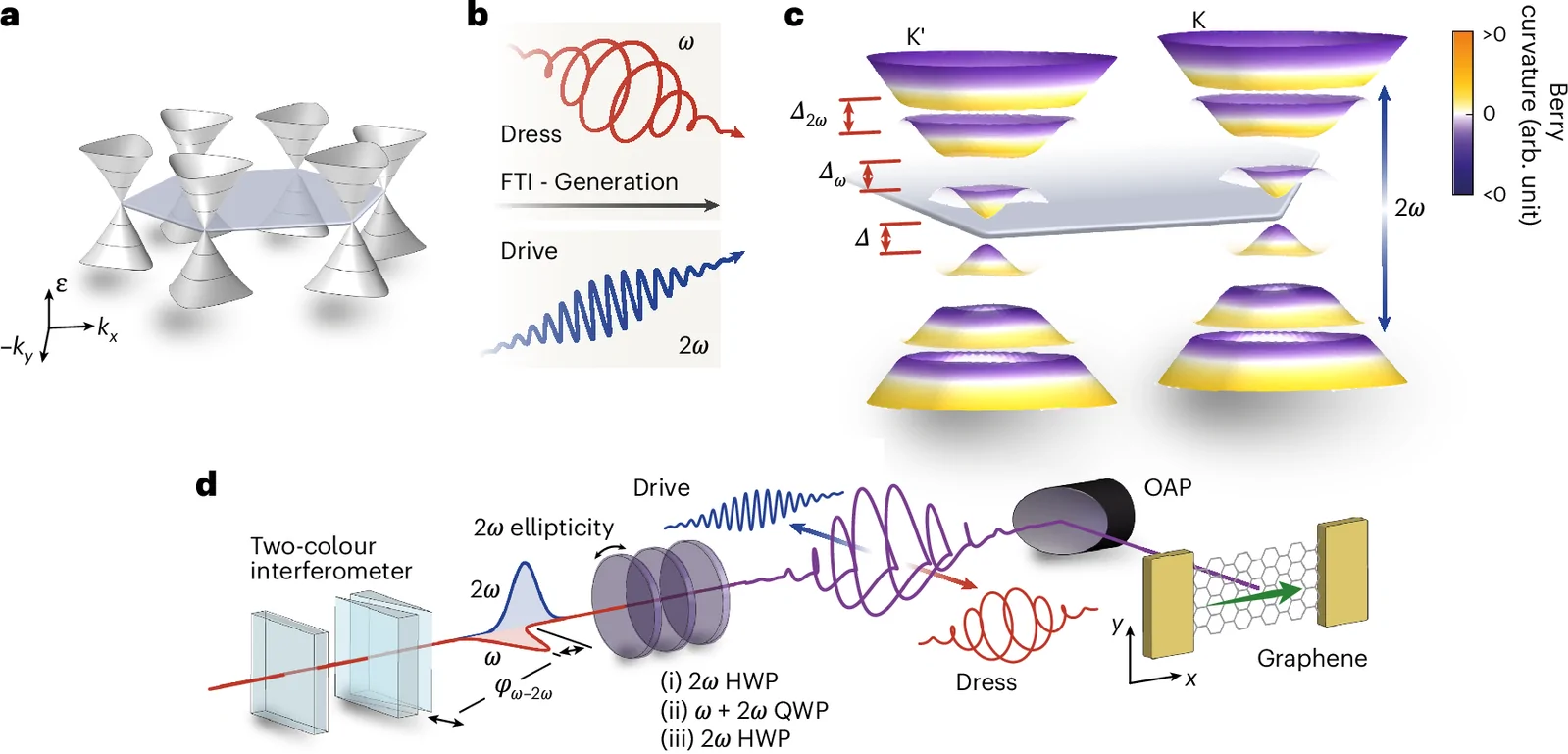
Lightwave control of electrons in an FTI. **a**–**c**, An FTI emerges from driving the gapless, topologically trivial band structure of graphene (**a**) by irradiation with a circularly polarized fundamental laser field (**b**, red waveform). This dressing breaks only time-reversal symmetry, giving rise to both a non-zero gap *Δ* at the emerging K and K′ valleys and a Berry curvature (colour bar) of the same sign in both valleys, resulting in a non-zero Chern number and a non-trivial topological insulating phase, with avoided crossings at resonant energies (*Δ*_*nω*_) (**c**; see Supplementary Fig. 2 for details). A second-harmonic pulse (**b**, blue waveform) controls the motion of electrons in the FTI bands. We probe CD and the all-optical anomalous Hall effect via photocurrents as signatures of the Berry curvature in the Floquet insulating phase. **d**, Sketch of the experimental set-up. The phase *φ*_*ω*−2*ω*_ between the copropagating *ω* and 2*ω* pulses is controlled in a collinear two-colour interferometer. In a sequence of dichroic half-wave plates (HWP) and a superachromatic quarter-wave plate (QWP; Methods) we shape the *ω* circular dress and the 2*ω* drive pulse with arbitrary polarization. Using an off-axis parabolic mirror (OAP), we focus the resulting waveform on a monolayer graphene strip, mounted in a vacuum chamber (not shown). We measure *φ*_*ω*−2*ω*_- and polarization-dependent photocurrents via gold electrodes attached to the graphene strip.

To generate an FTI, we optically dress bare graphene (Fig. 1a) with a circularly polarized fundamental laser field (with carrier angular frequency *ω*; Fig. 1b). This opens topological bandgaps within the dressed band structure (Fig. 1c). In this transient topological band structure, a second ‘drive’ laser pulse (with carrier angular frequency 2*ω*; Fig. 1b) drives coherent ultrafast electron dynamics in the light-dressed state, generating photocurrents.

While traditional measurements of transport phenomena in FTIs are done with d.c. or low-frequency fields, here we use an all-optical approach, which will be critical for elucidating new phenomena in solid-state FTIs. Our FTI exhibits avoided crossings capable of topological phenomena^18^ at harmonics of the dressing energy (calculated band structure colour coded with Berry curvature, *Ω*(**k**), *Δ*_*nω*_; Fig. 1c). The second harmonic drives transitions at the topological bandgaps, *Δ*_*nω*_. The fundamental field’s helicity and strength play the dominant role in defining the topological state (and Berry curvature) of our system^18,19^ allowing us to utilize our harmonic Floquet spectroscopy technique.

By optically modifying the material with the dressing light and subsequently driving electrons with the second harmonic, we are able to perform two unique measurements. First, the micromotion (the sub-optical-cycle motion of the Floquet state resulting in attosecond phenomena) can be directly mapped onto the two-colour phase, *φ*_*ω*−2*ω*_, the phase difference between *ω* and 2*ω* light^4,8,20–23^ (Fig. 1d). Second, the helicity (that is, symmetry) of the second harmonic can be used to probe the symmetry of the Floquet state^8,21,24^, that is, the Berry curvature *Ω*(**k**) at the harmonic resonances (Fig. 1c, right). This is because the transition matrix elements for excitation within the FTI band structure simultaneously depend on the Berry curvature (imparted by 1*ω*), the helicity of 2*ω*, and the Floquet phase. With a highly hybridized FTI band structure, we expect excitation at harmonics of the dressing field (*nℏω*), exactly where Berry curvature is imparted by the dressing field. By independently varying the ellipticity and *φ*_*ω*−2*ω*_ of the second harmonic, we can visualize quantum phenomena arising from the transient light-dressed FTI state stroboscopically, taking full advantage of harmonic Floquet spectroscopy.

Experimentally, we measure optical waveform-dependent photocurrents resulting from the two-colour set-up for different ellipticities and relative phases. The optical set-up used for the synthesis of the two fields is sketched in Fig. 1d. Part of the light from an 80-MHz femtosecond erbium fibre laser is frequency doubled to form 2*ω* pulses with a photon energy of 1.6 eV (775 nm, 110 fs intensity full width at half maximum, FWHM), which we overlap in a highly stable collinear set-up with the fundamental *ω* pulses at 0.8 eV (1,550 nm, 213 fs FWHM). The relative phase *φ*_*ω*−2*ω*_ between the pulses is controlled by calcite wedges in the collinear two-colour interferometer^25^. After polarization control with a sequence of dichroic wave plates, the pulses are focused onto epitaxial monolayer graphene on silicon carbide using an off-axis parabolic mirror. The resulting currents are measured via attached gold electrodes. The fundamental field strength at the sample focus is measured to be in the strong-field regime at 0.27 V nm^−1^, with a 3:4 field ratio of second harmonic to fundamental (equivalently, a ratio of 3:8 in vector potential; Methods). The frequency choice for the dressing and drive pulses results in short optical cycles avoiding decoherence^26^ and excitation well above the Fermi level of graphene on SiC (~300 meV)^17^, placing us in the high-frequency driving limit^27^. Specifically, the optical driving period of ~5 fs is faster than electronic decoherence time in graphene^28^, allowing us to generate and probe pre-thermal nonequilibrium states before decoherence and thermal relaxation take hold^27^. If and how dissipation from graphene to SiC plays a role must be left to future research. Importantly, dressing with strong near-infrared fields still allows for relevant gap sizes to open (a 53-meV bandgap at K/K′ is theoretically expected; see Supplementary Text for more information) while taking advantage of mature, high-power and low-noise optical sources^29^. This results in a Floquet parameter^16^ ${{\mathcal{F}}}=\frac{ea{E}_{\omega }}{\hslash\omega}$ of 0.08 (where *e* is the electron charge, *a* is the lattice constant, *E*_*ω*_ is the dressing field strength and *ℏ* is the reduced Planck’s constant). Our Floquet parameter, gap sizes and electric field (and vector potential) suggest that we are within the strong field regime^13^. We note that graphene sits at the edge of the Haldane phase diagram^30^, such that any circular dressing renders the system topological.

## Circular dichroism with an FTI

**Fig. 2:**
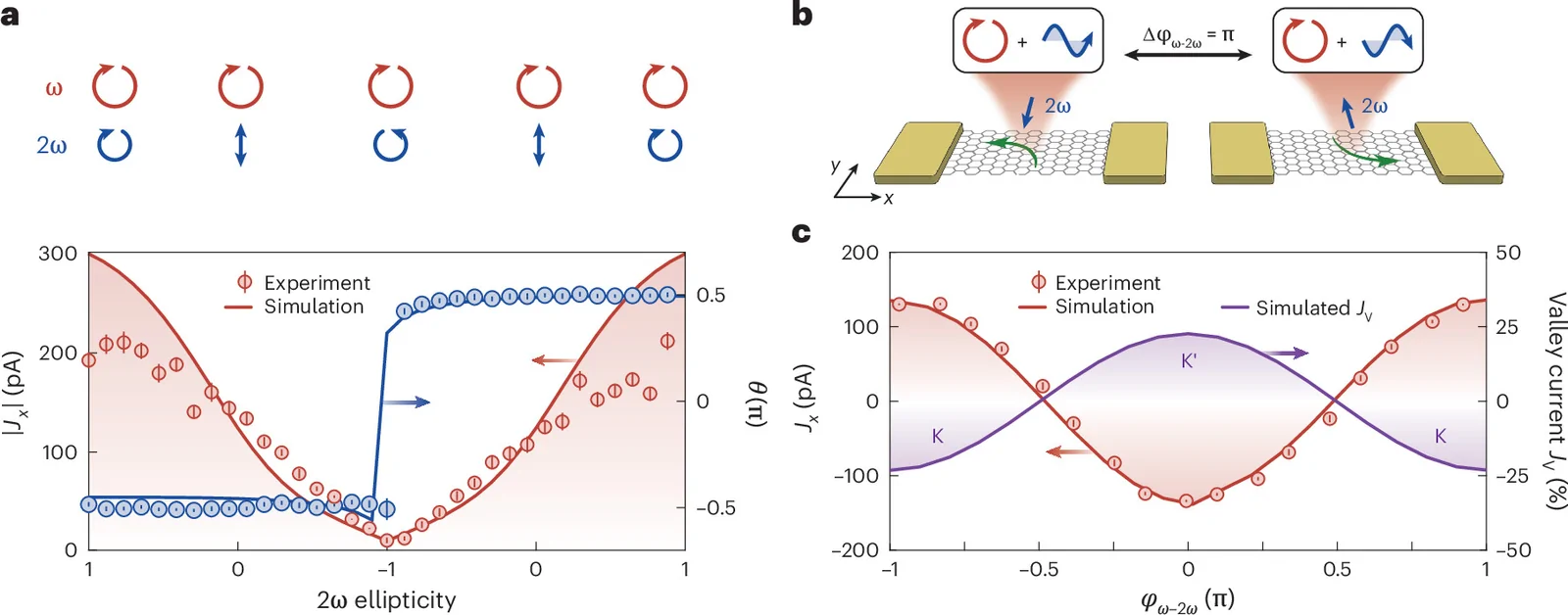
CD and all-optical anomalous Hall effect. **a**, We probe CD by keeping the helicity of *ω* constant, generating the FTI, and continuously sweeping the ellipticity of 2*ω* (top). Bottom: measured current amplitude ∣*J*_*x*_∣ (red circles) and phase *θ* (blue circles) as a function of the 2*ω* ellipticity. TDDFT simulation results (lines) support the dichroic generation of ballistic photocurrent for co-rotating helicities of *ω* and 2*ω* and suppression for counter-rotating helicities. **b**, All-optical anomalous Hall currents (green arrows) emerge from excitation of the *ω*-driven FTI with 2*ω* control polarized perpendicularly to the electrode axis. Flipping *φ*_*ω*−2*ω*_ by π effectively results in a reversal of the 2*ω* deflection and consequently also the Hall current direction. **c**, Measured current (red circles) as a function of *φ*_*ω*−2*ω*_. The computed current density (red line) confirms the directional control of the anomalous Hall effect. The computed degree of valley-polarized current *J*_V_ (purple line) reaches nearly 25%, realizing preferential current generation from either K or K′. For the ab initio calculations, pulses at 1,550 nm with *E*_0_ = 0.27 V nm^−1^ are used with 75% 2*ω* field admixture. For all plots, circles represent the mean of 83 experimentally measured photocurrents with error bars of ten times the standard deviation; solid lines indicate calculated photocurrents from TDDFT. Source data

We first analyse circular dichroism (CD) in photocurrent responses. That is, we explore the system’s helicity-dependent response, which arises as a direct manifestation of its Berry curvature^31,32^. This is a probe of the broken symmetry (and topological property) of our FTI state (Fig. 2a). Importantly, graphene naturally does not display a CD response owing to both the inversion and time-reversal symmetry^24^, resulting in zero Berry curvature. Once graphene is dressed by the purely circular *ω* dressing field, the resulting FTI state only breaks time-reversal symmetry^1,24^, enabling a CD response. The two-colour approach allows us to visualize this time-reversal symmetry broken state explicitly because the excitation pathways are probed within the FTI by the excitation (2*ω*) light. These pathways are dependent on both the Berry curvature of the FTI state (arising only from broken time-reversal symmetry by the dressing field, *ω*) and the helicity of the excitation light (2*ω*). Based on the periodicity of the light fields used, we expect conduction band population capable of generating *k*-space asymmetries leading to photocurrents to occur at harmonics of the fundamental field (shown schematically in Fig. 1c). Importantly, the first two avoided crossings exhibit topological phenomena (that is, non-zero Berry curvature), similar to the opened gap at the Dirac point^18^. When the helicity of the 2*ω* probe is matched to the Berry curvature of the FTI band structure, photocurrents should be generated (momentum imbalance of conduction band population); and when the 2*ω* helicity is antimatched to the Berry curvature, a suppression of photocurrents should occur^14,24,31^. This photocurrent CD may be understood as the *k*-space-resolved counterpart of optical CD. When integrated over the Brillouin zone, our observable directly measures the imaginary part of the optical (a.c.) Hall conductivity^32^.

We experimentally observe this photocurrent suppression by nearly two orders of magnitude as the 2*ω* helicity goes from *ϵ* = 1 to *ϵ* = −1, while the *ω* helicity is fixed to *ϵ* = 1, preserving the sign of *Ω* (Fig. 1c). These results are well matched by ab initio time-dependent density functional theory simulations (TDDFT, full line in Fig. 2a; Methods). We stress that our photocurrent CD is obtained by reversing the helicity of the drive (2*ω*), and not that of the dressing (*ω*) field. This is fundamentally different from pump CD exhibited in inversion-asymmetric materials, where the helicity of the dressing (*ω*) light couples to only one of the valleys in a gapped system^33,34^. Indeed, this CD is a direct result of the broken time-reversal symmetry within the Floquet Chern insulating state, leading to an actual change in photocurrent magnitude, not only a sign flip. Hence, our work represents a measurement of the FTI state’s intrinsic non-trivial dichroic response, directly enabled by the optical approach we use.

## All-optical anomalous Hall effect in an FTI

Next, we investigate the ultrafast motion of electrons driven by a now linearly polarized drive pulse in the FTI bands. A key hallmark of the FTI phase is the appearance of the anomalous Hall effect^1,13,17^ (schematically shown in Fig. 2b), where a current is measured orthogonally to an applied voltage. This effect stems from the same-signed Berry curvature (*Ω*) of the conduction band for both K/K′ valleys causing a deflection of electrons driven linearly by the second harmonic, **j** = −*ρ*^c^(*e*/*ℏ*)**E**_2*ω*_ × *Ω* (with excited population *ρ*^c^ and second-harmonic driving field **E**_2*ω*_). This motion is similar to the Lorentz force for charged particles, but exerted in *k*-space. Here, we orient the linear polarization of the second harmonic in the *y*-direction and measure *φ*_*ω*−2*ω*_-dependent currents along *x* (Fig. 2b,c). In a complete mapping of the photocurrent as a function of the two-colour phase (Fig. 2c), we observe a reversal of the current between *φ*_*ω*−2*ω*_ = 0 and π. This shows that the optical phase controls the anomalous Hall current direction and hence maps the sub-optical-cycle, attosecond, micromotion of the FTI state^8,20^. We therefore demonstrate an attosecond measurement analogous to microsecond motion in synthetic cold atom lattice systems^11,12^, but in a real solid enabled by using atomically relevant fields and carrier frequencies. Most importantly, this represents an all-optically induced anomalous Hall effect measurement, in graphene, coherently controlled by two-colour attosecond subcycle optical delay.

Ab initio TDDFT simulations that do not include edge states (Fig. 2a,c) agree remarkably well with the experiment (see Methods for details). This implies that we can rule out the contribution of edge states in these experimental conditions and that topologically protected states are not the main contributor to conductance, rather bulk states. Further analysis of TDDFT results suggests that the primary mechanism is from Floquet physics, due to the majority role of the first valence and conduction bands of graphene, the alignment of population with dressed bands, and the lack of observed electron–electron interactions (see discussion in Methods).

Interestingly, the excellent agreement of experiment and theory allows us to unravel the origin of the currents by decomposing them into contributions from the K and K′ valleys. This analysis shows that the photocurrent is ~25% valley selective (Fig. 2c, purple line, 31 pA maximum), with one valley carrying a larger population imbalance (Methods). Despite the energetically indistinguishable K and K′ valleys, broken time-reversal symmetry in the FTI valleys allows graphene to be harnessed for valleytronics^35^.

## Unravelling the Berry curvature photocurrent enhancement

Finally, we resolve the role of the Berry curvature of the FTI state in the photocurrent generation using ab initio simulations of the light–matter interaction. Here, we can compare two extremes: one where the light–matter symmetry precludes Berry curvature effects, and one where they should be present.

**Fig. 3:**
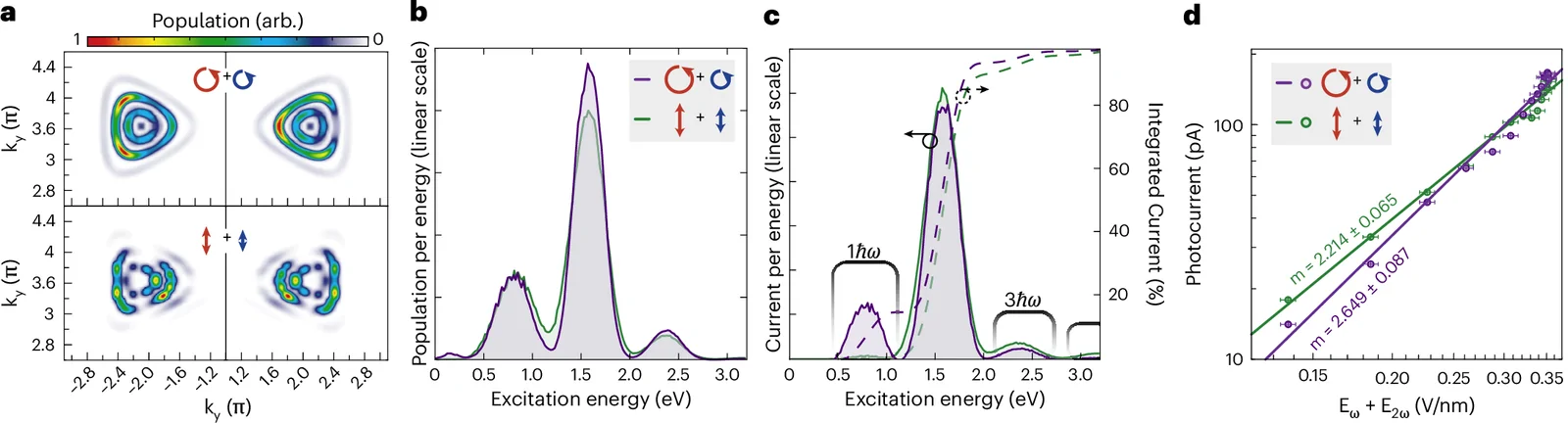
Extracting the role of the light-induced Berry curvature. To understand the role of the Berry curvature in the photocurrent generation, we compare the photocurrent generation from the co-rotating waveform and a linearly polarized bichromatic field. **a**, Ab initio calculated momentum fingerprints of the conduction band population from the co-rotating waveform (top) and a co-linearly polarized waveform (bottom), showing the population distribution in *k*-space. **b**, Population of the conduction band as a function of excitation energy for the two waveforms, showing similar distributions of electrons. **c**, Excitation energy-resolved photocurrents for the two fields, showing a clear difference in current contributions for the one-photon resonance (1*ℏω*). Integrated photocurrent contributions (dashed curves) are displayed on the right axis. The one-photon resonance contributes 17% of the integrated current for the co-rotating case, and with <1% in the co-linear case. Higher-order (>2*ℏω*) resonances account for 5.4% and 8% of the photocurrent signal for the co-rotating and co-linear cases, respectively. **d**, Power-scaling for both the co-linear and co-rotating cases show non-perturbative effects, with slopes of 2.214 ± 0.065 and 2.649 ± 0.087, respectively; circles indicate average *φ*_*ω*−2*ω*_-dependent photocurrent (2,490 samples), and error bars indicate the 1*σ* fit uncertainty. The slopes are different by 4.0*σ*. The additional photocurrent contribution at 1*ℏω* for the co-rotating case results in a stronger nonlinearity, seen in **d**, and can be understood by the Berry curvature of the FTI state, which is absent in the co-linear case. Source data

We first compare normalized conduction band populations for two sets of two-colour waveforms (Fig. 3a), with different polarization states. Both two-colour waveforms generate photocurrents, the co-rotating in the *x*-direction, and the co-linear in the *y*-direction only. Unsurprisingly, the conduction band population plotted versus energy (Fig. 3b) reveals similar distributions for the two waveforms due to the similar field strengths. The difference between the two cases arises when taking the Fermi velocity of the bands to calculate the energy-resolved photocurrent (Fig. 3c).

We see that both cases also contain substantial populations above the dominating two-photon resonance (labelled 3*ℏω*, and higher), with 8% for the co-linear case and 5.4% for the co-rotating case, indicating that non-perturbative physics is at play. A strong contribution of 17% of the total photocurrent arises from the one-photon resonance (1*ℏω*) in the case of the co-rotating waveform. Strikingly, this photocurrent contribution is absent from the co-linear waveform (<1% of the total photocurrent).

Before we discuss this further, we highlight photocurrent power scaling measurements for the two cases shown in Fig. 3d. We observe most clearly that the nonlinearities for the two cases differ substantially: 2.649 ± 0.087 for co-rotating driving and 2.214 ± 0.065 for co-linear, different by 4.0*σ*. We note that the exponent may vary with field strength, although limited data precludes a well-constrained multislope fit (Methods). The scan preserves the *E*_2*ω*_:*E*_*ω*_ ratio throughout, such that a purely perturbative process would give *m* = 3. This establishes that the observed exponents reflect a genuine departure from typical perturbative scaling.

The large photocurrent contribution present at the one-photon resonance can be intuitively understood through the use of FTI physics and has direct impact on the power scaling. Here, our circular field dresses graphene, strongly hybridizing the band structure and breaking time-reversal symmetry, generating same-signed Berry curvature at the one-photon resonance around both K and K′ (Supplementary Fig. 2). This Berry curvature enables previously symmetry-forbidden transitions to occur resulting in asymmetric conduction band populations^24,36^. The co-linearly polarized dressing field, by contrast, preserves the symmetry that forbids this transition pathway, so no analogous 1*ℏω* enhancement occurs in that case. Following a similar formalism as in ref. ^37^, we can write the helicity-dependent conduction band population as

1 $${n}_{c}^{{\sigma }^{\pm }}({\bf{k}})\propto \left[\frac{1}{2}\left(| {r}_{cv}^{x}{| }^{2}+| {r}_{cv}^{y}{| }^{2}\right)\pm \frac{i}{2}\left({r}_{cv}^{\,x}{r}_{vc}^{\,y}-{r}_{cv}^{\,y}{r}_{vc}^{\,x}\right)\right]\delta \,\left(\hslash \omega -{\varepsilon }_{cv}({\bf{k}})\right),$$

where ${r}_{ij}^{xy}$ are the conduction-to-valence band *x* and *y* transition dipole matrix elements, and *δ* is the Kronecker delta function. The first set of dipole matrix elements, $\frac{1}{2}\left(| {r}_{cv}^{x}{| }^{2}+| {r}_{cv}^{y}{| }^{2}\right)$, is symmetric with respect to *k* and, hence, does not lead to photocurrent generation. The second set, $\frac{i}{2}\left({r}_{cv}^{x}{r}_{vc}^{y}-{r}_{cv}^{y}{r}_{vc}^{x}\right)$, are the antisymmetric cross terms resulting in photocurrent generation. Intriguingly, this term is directly equivalent to the Berry curvature, as by definition in a Bloch two-band system: $i\left({r}_{cv}^{x}{r}_{vc}^{y}-{r}_{cv}^{y}{r}_{vc}^{x}\right)={\Omega }_{cz}({\bf{k}})$ (see equation 1.27 in ref. ^36^). This connects the local Berry curvature, present for the co-rotating case and absent from the co-linear case, to an additional photocurrent pathway. Importantly, this is experimentally corroborated by the different power scalings.

It is worth noting that, while a standard two-colour coherent control approach^38,39^ could capture the main two-photon contribution, it cannot capture the full picture of both the co-linear and co-rotating photocurrent physics discussed here, specifically the one-photon photocurrent contribution and the non-perturbative scaling. Perhaps higher-order nonlinear mixing terms or a time-dependent perturbation theory leading to a.c. Stark-shifted terms^40^ could explain the photocurrents, but neither provides an intuitive and unified view of the data. By contrast, the Floquet analysis (routine today in atomic physics^8^) can do so rather insightfully. A comparison with prior 800-nm coherent-control work^28^ is given in Supplementary Text.

## Optical electron control within the FTI

**Fig. 4:**
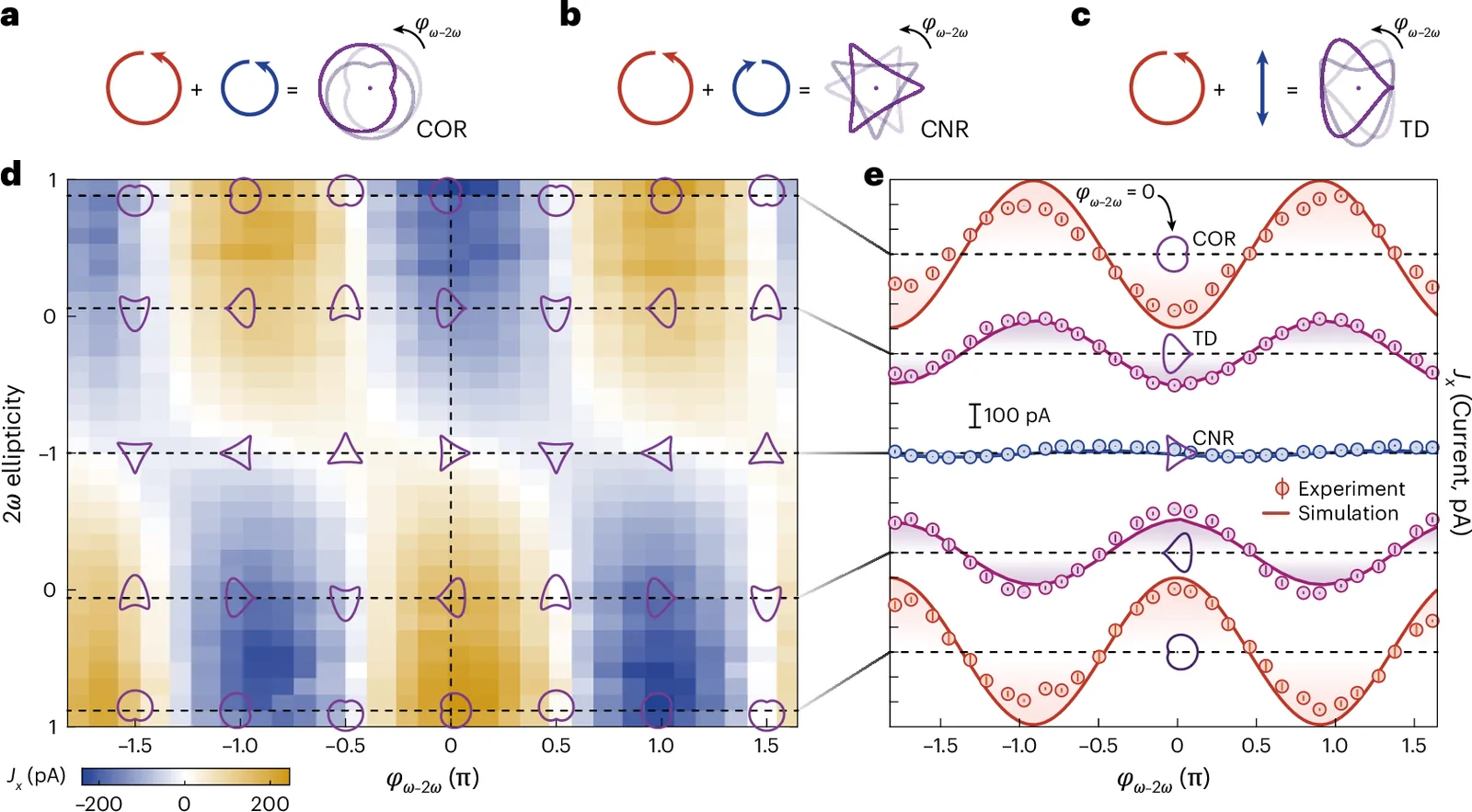
Relative phase and polarization control over measured photocurrents. **a**–**c**, For clarity, we highlight three examples of the superposition of *ω* + 2*ω* fields (Lissajous figures) resulting in co-rotating circularly polarized (COR, **a**), counter-rotating circularly polarized (CNR, **b**) and teardrop-polarized (TD, **c**) waveforms, with variable orientation determined by the two-colour phase (*φ*_*ω*−2*ω*_). **d**, Two-dimensional map of relative phase-dependent current as a function of *φ*_*ω*−2*ω*_ and the 2*ω* ellipticity. The insets exemplify the Lissajous figure transformation. **e**, Lineouts (horizontal dashed lines in **d**) as a function of *φ*_*ω*−2*ω*_ indicating the generation of current (red, COR), valley current (purple, TD) and valley polarization (blue, CNR) in the absence of current. Circles represent the mean of 83 experimentally measured photocurrents with error bars of ten times the standard deviation; solid lines indicate calculated photocurrents from TDDFT. Source data

We next explore photocurrent generation in the topologically non-trivial graphene FTI, measuring all combinations of drive (2*ω*) ellipticity and *φ*_*ω*−2*ω*_ with a fixed dressing field (Fig. 4). To aid clarity for both the evolving 2*ω* ellipticity and *φ*_*ω*−2*ω*_, we utilize Lissajous figures; three examples are shown in Fig. 4a–c. These Lissajous figures result from the superposition of the fundamental laser field (*ω*) and its second harmonic (2*ω*) with properly chosen polarization states and *φ*_*ω*−2*ω*_ (Fig. 1d).

With matching fundamental and second-harmonic helicities, a co-rotating circularly polarized (COR) waveform emerges (Fig. 4a). As discussed previously, the matched symmetries (that is, helicity of 2*ω* and Berry curvature *Ω*) result in strong photocurrent generation. Interestingly, the photocurrent scaling for the COR case has a higher nonlinearity than that for co-polarized linear fields (Supplementary Text), suggesting an added contribution to the current from the Berry curvature of the FTI state^13^. Changing *φ*_*ω*−2*ω*_ results in the 2*ω* drive field sampling a different phase of the FTI micromotion, resulting in a reversal of the photocurrent. Similarly, the Lissajous figure exhibits a dynamical (that is, time-periodic) symmetry plane in one direction that rotates upon changing *φ*_*ω*−2*ω*_ (ref. ^41^). With opposite helicities of fundamental and second harmonic, a counter-rotating circularly polarized (CNR) waveform emerges and exhibits a threefold rotational dynamical symmetry^41^ (Fig. 4b). As shown in the CD measurement (Fig. 2a), this 2*ω* helicity results in photocurrent suppression. By tuning *φ*_*ω*−2*ω*_ the Lissajous figure rotates. This waveform has been studied extensively for producing inter-valley population imbalances, that is, valley polarization^35,42–45^. While our FTI state (with only broken time-reversal symmetry) has energy-equivalent K/K′ points, the non-equivalent orbital character of K/K′ allows us to use *φ*_*ω*−2*ω*_ to address them differently. This FTI state is fundamentally different from systems with broken inversion symmetry where the K/K′ points are energy-inequivalent. Finally, the last case with a linear 2*ω* drive field is shown in Fig. 4c. As *φ*_*ω*−2*ω*_ changes, the Lissajous figure continuously deforms and rotates the combined field’s symmetry axis. Revealed by ab initio simulations, this teardrop (TD) waveform leads to a current dominantly arising from one valley only, that is, a valley current.

To experimentally investigate these three distinct cases, we map out photocurrents as a function of all relevant 2*ω* polarization states and phases (Fig. 4d). On the vertical axis, as we tune the 2*ω* ellipticity while keeping *ω* circular, we continuously sweep from a COR to a TD and a CNR waveform and further to a TD and COR waveform again. As the fast and slow axes of the 2*ω* half-wave plate are interchanged by sweeping across ellipticity −1, a π phase jump is introduced between the two colours, resulting in an inversion of current direction with approximately identical amplitudes for equal ellipticities >−1. A notable magnitude of up to 214 pA is obtained for the COR waveform and can be understood from the increased photocurrent contribution of the *ℏω* resonance and its associated Berry curvature. As the wave plate is rotated to generate the TD waveform, ∣*J*_*x*_∣ decreases to approximately 140 pA. Finally, with the CNR waveform, ∣*J*_*x*_∣ further decreases to a minimum of 12 pA. Importantly, the strong overall suppression of current injection supports the generation of valley polarization by the bichromatic waveforms (Supplementary Text).

Changing *φ*_*ω*−2*ω*_ with the two-colour interferometer results in a continuous change of the Lissajous figures, which we observe experimentally as a sinusoidal oscillation of injected *x*-direction current (see Fig. 4d as well as lineouts and solid circles in Fig. 4e). This sinusoidal behaviour of the photocurrent maps the Floquet state micromotion^12,20,21,23^. The vertical lineout for *φ*_*ω*−2*ω*_ = 0 as well as the horizonal lineout for 2*ω* ellipticity of 0 are also presented in Fig. 2a,c, respectively. Overlaid on the experimental data is the calculated current from TDDFT (normalized to the TD waveform current amplitude; Methods). We observe similar behaviour between the experiment and theory, as well as a small non-zero current for the CNR waveform with a phase-shifted maximum, showing remarkable agreement with the measurement. Interestingly, this agreement for the CNR waveform suggests a spontaneous breaking of the temporal symmetry for the excitation, meaning that, as the valley polarization builds up, it becomes more challenging to excite electrons to the conduction band. TDDFT simulations rule out correlated electron effects (Supplementary Text) as a source of this feature.

## Symmetries, selection rules and subcycle dynamics

For further insight, it is highly instructive to investigate how the *ω*−2*ω* joint light field symmetries are reflected in the excited state populations. Critically, these symmetries will allow us to understand when photocurrent generation is allowed or forbidden (but will never predict the magnitude and scaling, as part of the current will originate from the Berry curvature of the FTI state). This analysis can therefore connect the sub-optical-cycle electron motion in the undressed band structure to the topological phenomena and micromotion present in the FTI picture, complementing the Floquet band picture.

**Fig. 5:**
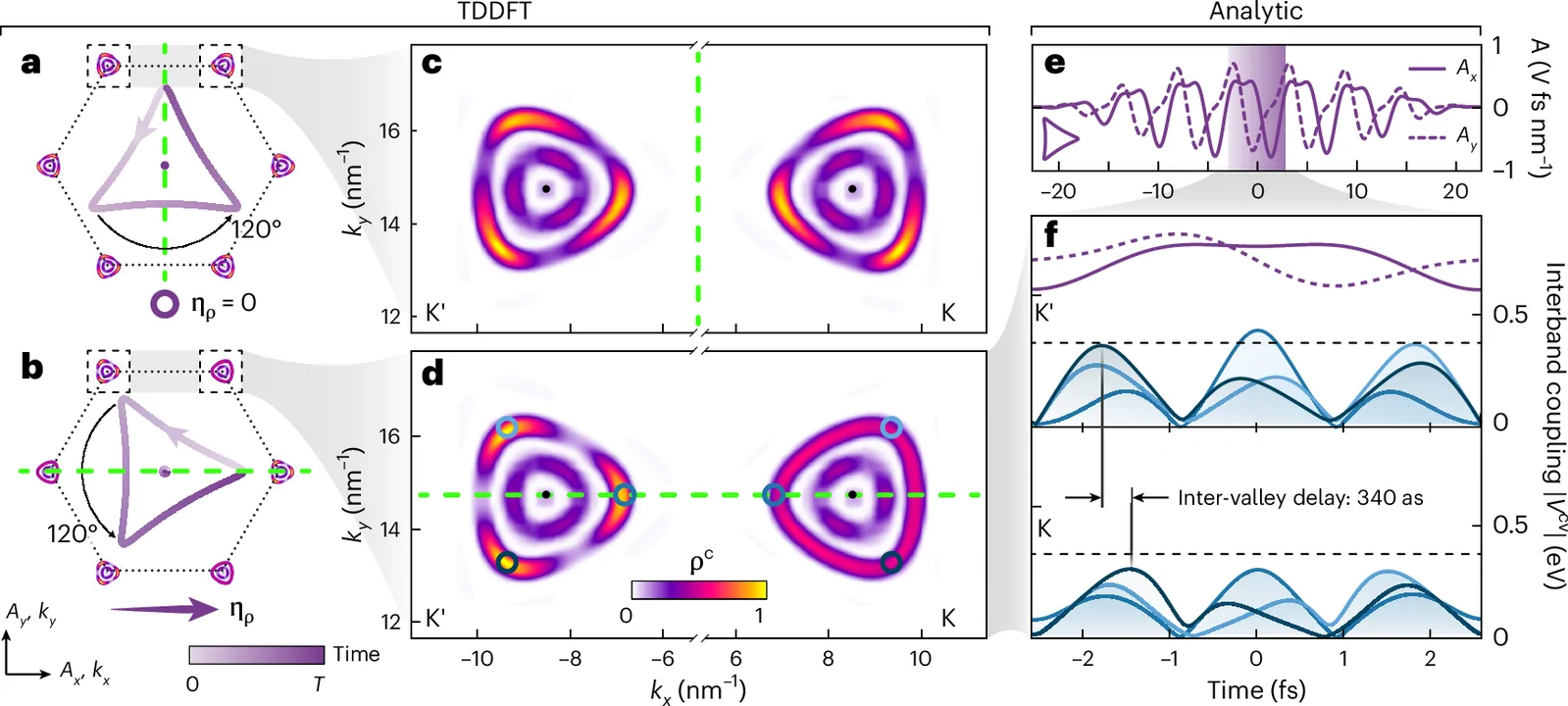
Subcycle dynamical symmetry selection rules. **a**,**b**, Temporal shape and symmetries of the vector potential Lissajous figures (purple lines) and the resulting symmetries of residual momentum distributions in the conduction band (*ρ*^c^) imprinted to the first Brillouin zone (dotted hexagons) for the CNR waveforms for *φ*_*ω−2ω*_ = π/2 (**a**) and 0 (**b**), respectively. The momentum distributions are computed with TDDFT (Methods). Dashed green lines indicate global symmetry axes, determined by both waveform and time periodicity (see purple colour bar). **c**,**d**, Close-ups of *ρ*^c^ around K and K′ as indicated in **a** and **b**. For all cases, an eight-cycle pulse (vector potential shown in **e**) is applied with 1,550-nm fundamental wavelength with a peak field strength *E*_0_ = 0.27 V nm^−^^1^ and 75% 2*ω* field admixture. **e**, CNR vector potential waveform for *φ*_*ω*−2*ω*_ = 0. The shaded area indicates the centre optical cycle. **f**, Temporal profile of the interband dipole coupling ∣*V*^cv^∣ (ref. ^47^) induced by the waveforms shown in **e**. The different shades of blue refer to initial momenta in the K and K′ valley as marked with circles in **d**. The dashed black guide lines compare the ∣*V*^cv^∣ amplitudes at K and K′.

Figure 5a–d shows the residual conduction band distributions for the CNR waveform obtained from TDDFT (Methods). Analysing all six K and K′ points (Fig. 5a,b) allows us to look at the dynamical (periodic) symmetry selection rules, while the zoom-ins (Fig. 5c,d) highlight the intravalley symmetries^41,46^. Two symmetries dominate: (1) a mirror axis coupled to time-reversal in all geometries (dashed green lines), and (2) a threefold rotational symmetry coupled to time translations in the CNR case only. These symmetry operators map onto the momentum distributions of electrons in the conduction band. From these symmetries, we can assign selection rules to how the light field symmetries dictate phenomena such as valley polarization and valley current, representing an experimental mapping (Fig. 4d) of these dynamical symmetry selection rules.

We analyse these dynamical selection rules by looking at the momentum distributions (Fig. 5a–d) computed for the conditions where the measured photocurrent is suppressed (Fig. 4d), that is, where injection current is symmetry-forbidden. For the CNR waveforms (Fig. 5a,b), we see one mirror axis present in the electron excitation distributions: an M–Γ–M symmetry axis for *φ*_*ω*−2*ω* _= π/2 (Fig. 5a, dashed green line), rendering the conduction band population (*ρ*^c^) K–K′ symmetric, hindering the ability to generate valley polarization. When *φ*_*ω*−2*ω* _= 0, we disconnect the K and K′ points rotating the symmetry axis to K–Γ–K′ (Fig. 5b, dashed green line). This allows us to address K/K′ differently, and results in a non-zero valley polarization of *η*_*ρ*_ = 1% (Methods). The zoom-ins of the CNR population (Fig. 5c,d) reveal the local *ρ*^c^ symmetries within the K and K′ valleys. The total injection current should vanish due to the rotational dynamical symmetry in the CNR case (as currents from all K and K′ points individually cancel each other). Experimentally (and numerically in TDDFT) we observe a small residual current (Fig. 4e) that occurs from breaking of time-translational symmetry by the pulse envelope itself (that is, departing from an infinitely time-periodic Floquet Hamiltonian). The COR and TD waveforms are further analysed in Supplementary Text.

To understand the emergence of the characteristic *ρ*^c^ patterns in Fig. 5d on a subcycle time scale, it is insightful to further discuss the time-dependent inter- and intraband electron dynamics in the undressed band structure. We highlight these dynamics for three exemplary electrons with initial momenta equidistant to the respective K and K′ points (Fig. 5d, coloured circles). These electrons are chosen to show the best comparison between all three waveforms showcased in this Article (see Supplementary Information for the other waveforms). Figure 5f shows the respective subcycle evolution of the interband dipole coupling *V*^cv^(*t*) = **E**(*t*) ⋅ **d**^cv^ (instantaneous Rabi frequency divided by *ℏ*), which approximates the instantaneous excitation probability for a given electric field **E**(*t*) and an interband transition dipole matrix element **d**^cv^(**k**(*t*)) (ref. ^47^). The stark intervalley differences in ∣*V*^cv^∣ occurring during the centre optical cycle (Fig. 5e, shaded area) become clear with the corresponding traces drawn in Fig. 5f. The differences in the subfemtosecond trajectories of electrons between K and K′ show how the composite light field can be used to understand the valley-selective physics. By looking at the coupling strength peaks, we observe intervalley population differences forming within a time delay of 340 as between two *k*-space mirrored points. Unsurprisingly, these dynamics are identical to 2*ω*-driven electrons sampling the phase-dependent micromotion of the *ω*-induced topological FTI band structure, indicating the duality of the two physical pictures.

## Conclusion and outlook

We have established harmonic Floquet spectroscopy—a new probe for non-thermal, ultrafast excitations—and used it to control electrons in light-dressed graphene, generating photocurrents that provide compelling evidence for their Floquet-topological origin. We measured the all-optical anomalous Hall effect, photocurrent CD and a Berry curvature photocurrent enhancement utilizing our two-colour optical set-up. The photocurrents show an exceedingly strong phase and ellipticity dependence, allowing us to use them to probe the ultrafast dynamics in the FTI. Excellent agreement with ab initio theory allowed us to pinpoint the source of currents to the FTI valleys at K and K′ and to rule out involvement of topologically protected edge states. This further leads to a valley current (with a magnitude of 31 pA) from an FTI. For a specific case, we considered the interaction of the two-colour light field with the bare material, revealing experimentally measured lightwave dynamical symmetry photocurrent selection rules. This symmetry picture maps the sub-optical-cycle electron motion onto the topological photocurrents, showing the deep connection between these two distinct bases, the lightwave symmetries and the FTI states.

Beyond these specific measurements, our results point towards Floquet lightwave topotronics^48^ as a general strategy: topology imprinted by an optical dressing field, with electron motion steered by a second field on (sub-)femtosecond timescales. Our experiments showcase such control through photocurrent CD and the all-optical anomalous Hall effect, showing sub-optical-cycle control of these photocurrents and relating them to the attosecond micromotion inside the FTI. Future work will focus on understanding the role of dissipation and other material-related properties in extending functionalities of quantum dynamics far from equilibrium. Last, we note that the polarization state of the dressing field allows even more complex electron and band structure control^49^. We foresee direct ramifications in the realm of ultrafast quantum material science^27^, lightwave electronics^50^, topological quantum computing, sensing and ultrafast spectroscopy, even in conjunction with twistronics.

## Methods

### Experimental details

We use laser pulses at a fundamental photon energy *ℏω* = 0.8 eV (1,550 nm central wavelength) with a pulse duration of 213 fs (intensity FWHM) from an amplified 80-MHz erbium-doped fibre oscillator. After frequency doubling in a 1-mm-thin type-I phase-matched bismuth borate crystal (BiBO), fundamental and second harmonic (photon energy 2*ℏω* = 1.6 eV, 775 nm central wavelength, 110 fs FWHM) pulse pairs co-propagate with orthogonal linear polarization (Fig. 1d, red and blue envelopes).

A collinear two-colour interferometer based on birefringent calcite elements is used to control their relative phase *φ*_*ω*−2*ω*_ (Fig. 1d)^25^. The first *d*_a_ = 3-mm-thin calcite plate is oriented with its ordinary (o) axis parallel to the 2*ω* polarization and its extraordinary (e) axis parallel to *ω*; for the wedged calcite elements, the axes are rotated by 90° to invert the situation. As the two colours experience different refractive indices along the respective axes (${n}_{\omega }^{{\rm{e}}}=1.47$, ${n}_{\omega }^{{\rm{o}}}=1.64$, ${n}_{2\omega }^{{\rm{e}}}=1.48$, ${n}_{2\omega }^{{\rm{o}}}=1.65$), this configuration allows us to provide the *ω* pulses with a head-start after the first calcite plate, which are subsequently caught up by the 2*ω* pulses in the calcite wedge pair^51^. By finely tuning the inserted thickness *d*_b_ of the wedges, the relative phase is controlled as

2 $${\varphi }_{\omega -2\omega }=\frac{2\omega }{c}\left({d}_{{\rm{a}}}\,\Delta {n}_{\omega -2\omega }^{{\rm{oe}}}+{d}_{{\rm{b}}}\,\Delta {n}_{\omega -2\omega }^{{\rm{eo}}}\right),$$

with $\Delta {n}_{\omega -2\omega }^{kl}={n}_{\omega }^{k}-{n}_{2\omega }^{l}$. Temporal overlap is determined by maximizing the *ω* + 2*ω* sum frequency signal obtained from a type-II phase-matched beta barium borate crystal in a subsequent reference path. The collinear interferometer design enables a particularly high passive phase stability exhibiting 14.6 mrad integrated jitter in the angular stability of the bichromatic Lissajous figures over 1 h (Supplementary Fig. 1). Pulse dispersion upon wedge insertion is negligible as we sample fewer than four periods of *φ*_*ω*−2*ω*_ with rather narrow-band spectra at both colours.

For individual control over the ellipticity and rotation of the polarization ellipse of both colours, the delayed pulses are subsequently sent through a sequence of (1) a dichroic half-wave plate (Newlight Photonics) acting on 2*ω* only, (2) a superachromatic quarter-wave plate (Thorlabs) acting on both colours, and (3) a dichroic half-wave plate (Newlight Photonics, same as (1)), which counteracts the ellipsoidal tilt of the 2*ω* polarization introduced by wave plate (2) upon rotation of wave plate (1). As wave plate (1) is rotated (Fig. 1d), the ellipticity of the 2*ω* polarization is changed from +1 (half-wave plate angle 45°) to 0 (half-wave plate angle 22.5°) and finally −1 (half-wave plate angle 0°). Then, it is changed back to 0 (half-wave plate angle −22.5°) and finally +1 (half-wave plate angle −45°), while the *ω* polarization is kept constant at circular (ellipticity +1). This sequentially generates the COR, TD and CNR waveforms, and further the TD and COR waveforms with a phase slip of Δ*φ*_*ω*−2*ω*_ = π. We carefully calibrated the wave plates’ retardation to a wire-grid polarizer and ensured their dichroic performance with combined long- and shortpass filters. We measure a polarization extinction ratio of 14 dB for the combined harmonics (limited by the experimental detection method), corresponding to a residual orthogonal intensity fraction of 4%, that is, an ellipticity of 0.96 in the sense of the fraction of power in the intended polarization state. The resulting bichromatic waveforms are tightly focused onto a sample using a 15-mm off-axis parabolic mirror (Fig. 1d). We confirmed that neither rotation of the wave plates nor translation of the calcite wedges introduces observable changes in beam pointing or spot size at the focus, with beam-pointing deviations remaining below 1% of the beam radius for either motion.

We use a 2 × 10 μm strip of epitaxially grown monolayer graphene on silicon carbide as a sample. Its short sides are connected to 30-nm gold electrodes with a 5-nm titanium adhesion layer. Details on the fabrication and characterization are provided in ref. ^52^. Gold bond wires connect the electrodes with a chip carrier for photocurrent measurements. All measurements are performed under high vacuum conditions (10^−8^ hPa) at room temperature.

For photocurrent detection, we focus the pulse train to the centre of the structure such that only the graphene is illuminated. To isolate waveform-dependent currents from photo-thermoelectric currents and other static background, we modulate *φ*_*ω*−2*ω*_ from pulse to pulse at *f*_mod_ = 117 Hz with a piezoelectric actuator mounted to one of the calcite wedges. We amplify the resulting current via transimpedance amplification and record it via dual-phase lock-in detection referenced to *f*_mod_. Each data point shown in Fig. 4 is recorded with a 300-ms time constant and averaged over 25 s of data, resulting in an effective 83 measurements. The amplitude of the piezo stroke is calibrated via an external laser diode Michelson interferometer with one end mirror attached to the modulated calcite wedge. When the resulting modulation depth of *φ*_*ω*−2*ω*_ reaches π, the signal amplitude detected by the lock-in amplifier is maximized. The measured lock-in phase, *θ* (Fig. 2a), is equivalent to the absolute phase *φ*_*ω*−2*ω*_ as we determined the temporal overlap of both colours. The field-strength scan in Fig. 3d is recorded by attenuating the combined *ω* + 2*ω* beam with an achromatic attenuator placed downstream of the two-colour interferometer, so the attenuation acts identically on both colours and the *E*_2*ω*_:*E*_*ω*_ ratio is preserved at every point in the scan. Experimental errors from spot-size uncertainty, laser-power fluctuations and lock-in-amplifier noise are included in the quoted uncertainties; restricting the fit to a narrower range (for example, 0.1–0.3 V nm^−1^) does not meaningfully constrain the exponent given the limited data available there.

### Floquet calculations

We employ a Floquet Hamiltonian for the light-dressed graphene from a two-band nearest-neighbour tight-binding Hamiltonian

3 $${{\mathcal{H}}}_{0}({\bf{k}})=\gamma \left[\begin{array}{cc}0&f({\bf{k}})\\ {f}^{* }({\bf{k}})&0\end{array}\right],$$

with a hopping parameter *γ* = −2.9 eV and

4 $$f({\bf{k}})=\exp \left(i\frac{a{k}_{y}}{\sqrt{3}}\right)+2\exp \left(-i\frac{a{k}_{y}}{2\sqrt{3}}\right)\cos \left(\frac{a{k}_{x}}{2}\right),$$

where *a* = 2.46 Å is the lattice constant of graphene. By coupling ${{\mathcal{H}}}_{0}({\bf{k}})$ to the desired continuous-waveform **E**(*t*) of fundamental periodicity ${T}_{0}=\frac{2{\uppi }}{\omega }$ as ${\bf{k}}(t)={{\bf{k}}}_{0}+\frac{e}{\hslash }\int_{-\infty }^{t}{\bf{E}}({t}^{{\prime} }){\rm{d}}{t}^{{\prime} }$, we obtain the sub-blocks of the Floquet Hamiltonian as^53^

5 $${{\mathscr{ \mathcal H }}}_{{\rm{F}}}^{mn}({{\bf{k}}}_{0})=\frac{1}{{T}_{0}}{\int }_{0}^{{T}_{0}}{{\mathscr{ \mathcal H }}}_{0}({\bf{k}}(t)){{\rm{e}}}^{i(m-n)\omega t}{\rm{d}}t+{\delta }_{mn}m\hslash \omega {\sigma }_{0}.$$

∣*m* − *n*∣ is the order of the Floquet replica, which we numerically expand up to order 5. The replicas are obtained by diagonalizing ${{\mathscr{ \mathcal H }}}_{{\rm{F}}}$. We plot the resulting Floquet band structure in Supplementary Fig. 2a, where the colour weight is the overlap with the undressed band structure^1,13^. The calculated density of states (Supplementary Fig. 2b) shows open bandgaps at the K point, as well as the one-photon (*ω*) and two-photon (2*ω*) resonances. A quantized Hall effect requires a gap in the density of states, which may be realized in the two-colour experiment.

We directly calculate the Berry curvature as a summation of the eigenstates of the Floquet Hamiltonian

6 $${\Omega }_{\mu ,\nu }^{{\rm{n}}}({\bf{R}})=i\mathop{\sum }\limits_{{{\rm{n}}}^{{\prime} }\ne {\rm{n}}}\frac{\left\langle {\rm{n}}\right\vert \partial {{\mathcal{H}}}_{{\rm{F}}}/\partial {{\rm{R}}}^{\mu }\left\vert {{\rm{n}}}^{{\prime} }\right\rangle \left\langle {{\rm{n}}}^{{\prime} }\right\vert \partial {{\mathcal{H}}}_{{\rm{F}}}/\partial {{\rm{R}}}^{\nu }\left\vert {\rm{n}}\right\rangle -(\nu \leftrightarrow \mu )}{{({\epsilon }_{{\rm{n}}}-{\epsilon }_{{{\rm{n}}}^{{\prime} }})}^{2}}$$

due to the ability to avoid differentiation giving a freedom of gauge choice^36^. We plot the band structure in Supplementary Fig. 2c–f, where the colour encodes the curvature. Supplementary Fig. 2d–f shows the topological bandgap of 53 meV opened at the K point (Supplementary Fig. 2d), a 132-meV gap at the *ℏω* resonance (Supplementary Fig. 2e, 10× Berry curvature) and a 27-meV gap at the two-photon resonance (Supplementary Fig. 2f, 10× Berry curvature). It is evident that the circular driving imparts Berry curvature at the relevant avoided crossings of the band structure. The role of this added Berry curvature can be explored by scaling the bandgap size.

Finally, we can simulate the Floquet states (Supplementary Fig. 3) for experimentally achievable field strengths at 1,550 nm (refs. ^29,54^). Importantly, the shorter-wavelength dressing fields allow for substantially larger bandgaps (hundreds of milli-electronvolts) in graphene when compared with longer driving laser wavelengths. It is worth noting that these field strengths have already been used on graphene in previous work at 800 nm (ref. ^47^).

### Ab initio TDDFT simulations

We describe here details of the ab initio calculations used throughout the Article. All calculations are based on TDDFT simulations performed with the open access code Octopus^55^. We solved the time-dependent Kohn–Sham (KS) equations of motion, given in the velocity gauge and atomic units by

7 $$i{\partial }_{t}{\psi }_{n,{\bf{k}}}({\bf{r}},t)=\left(\frac{1}{2}{(-i{\boldsymbol{\nabla }}+{\bf{A}}(t)/c)}^{2}+{v}_{{\rm{KS}}}({\bf{r}},t)\right){\psi }_{n,{\bf{k}}}({\bf{r}},t),$$

where *ψ*_*n*,**k**_(**r**, *t*) is the KS–Bloch (KSB) state at band index *n* and *k*-point **k**, and *v*_KS_ is the KS potential that comprises the classical Hartree interaction term, the interactions of electrons with nuclei and deeper electronic states (which were described by norm-conserving pseudopotentials^56^), and the exchange-correlation term, where we used the adiabatic local density approximation. We have assumed partial periodic boundary conditions in the graphene monolayer plane (*xy*), while the *z* axis was treated with finite boundary conditions and a complex absorber of width 12 bohr during propagation (similar to ref. ^46^). In equation (7), **A**(*t*) is the applied vector potential after assuming the dipole approximation, which was taken to have the following form:

8 $$\begin{array}{l}{\bf{A}}(t)=f(t)c{E}_{0}\left(\displaystyle\frac{1}{\omega \sqrt{2}}(\cos (\omega t+{\varphi }_{\omega -2\omega })\hat{{\bf{x}}}+\sin (\omega t+{\varphi }_{\omega -2\omega })\hat{{\bf{y}}})\right. \\ \left. \qquad+\displaystyle\frac{1}{2\omega \sqrt{1+{\epsilon }^{2}}}(\epsilon \cos (2\omega t)\hat{{\bf{x}}}+\sin (2\omega t)\hat{{\bf{y}}})\right)\end{array}$$

where *c* is the speed of light, *E*_0_ is the electric field amplitude (taken at experimental values), *φ*_*ω*−2*ω*_ is the two-colour phase, *ϵ* is the ellipticity of the 2*ω* field, *ω* is the carrier frequency (taken at the experimental value) and *f*(*t*) is a temporal envelope (called a ‘super-sine’) taken as^57^

9 $$f(t)=\sin {\left(\pi \frac{t}{{T}_{{\rm{p}}}}\right)}^{\left(\frac{| \pi (\frac{t}{{T}_{{\rm{p}}}}-\frac{1}{2})| }{\sigma }\right)},$$

where *σ* = 0.75, *T*_p_ is the duration of the laser pulse, which was taken to be *T*_p_ = 8*T*, and *T* = 5.17 fs is a single cycle of the fundamental carrier frequency. This leads to an applied electric field of duration 20.6 fs (FWHM). In equation (8), by varying the relative phase and ellipticity, we obtain the waveforms plotted in the main text.

From the propagation of the KSB states, we obtained the current expectation value:

10 $${\bf{J}}(t)=\sum _{n,k}{w}_{k}\int\,\left[{\psi }_{n,{\bf{k}}}^{\dagger }({\bf{r}},t)\left(\frac{1}{2}(-i{\boldsymbol{\nabla }}+{\bf{A}}(t)/c)-i[{V}_{{\rm{ion}}},{\bf{r}}]\right){\psi }_{n,{\bf{k}}}({\bf{r}},t)\right]{\rm{d}}{\bf{r}}+c.c.,$$

where *V*_ion_ is the pseudopotential non-local part of *v*_KS_, *w*_*k*_ is the *k*-point weight, and the sum is performed over occupied states. From **J**(*t*), we calculated the actual measured current by averaging **J**(*t*) over a single cycle of the fundamental frequency after the driving laser pulse has ended (at *t* = *t*_*f*_): ${\bf{j}}=\int_{{t}_{f}}^{{t}_{f}+T}{\bf{J}}(t){\rm{d}}t$. We calculated the projection plots in Fig. 5 by projecting the time-dependent KSB states after the laser pulse (*t* = *t*_*f*_) has ended onto the ground states:

11 $${g}_{n}({\bf{k}})=\sum _{m}| \int\,{\psi }_{n,{\bf{k}}}^{*}({\bf{r}},0){\psi }_{m,{\bf{k}}}({\bf{r}},t_f){\rm{d}}{\bf{r}}{| }^{2},$$

where the sum runs over occupied states. From *g*_*n*_(**k**), we obtained band-resolved occupations, or summed over all conduction/valence bands as shown in Fig. 5. The final occupation plot was also interpolated on a denser *k*-grid and filtered.

The ab initio calculated phase in Fig. 2 was obtained from the injection current’s direction in the *xy* plane. The valley current in Fig. 2c was calculated by integrating separately for *k*_*x*_ > 0 and *k*_*x*_ < 0 regions in *k*-space in equation (11) for **J**(*t*), and using those quantities to define the normalized contribution of each half of the Brillouin zone:

12 $${J}_{{\rm{V}}}=\frac{| {{\bf{j}}}_{{\rm{K}}}| -| {{\bf{j}}}_{{{\rm{K}}}^{{\prime} }}| }{| {{\bf{j}}}_{{\rm{K}}}| +| {{\bf{j}}}_{{{\rm{K}}}^{{\prime}} }| },$$

with $|{{\bf{j}}}_{{\rm{K}}/{\rm{K}}^\prime}|$ being the injection current contributed from each region in the Brillouin zone.

We performed further auxiliary calculations where the time-dependent KS potential was frozen to its ground state form at *t* = 0, that is, *v*_KS_(**r**, *t*) = *v*_KS_(**r**, *t* = 0). This yields the independent particle approximation, where the different KSB states are decoupled from one another in the equations of motion and electron–electron interactions do not evolve dynamically. Supplementary Fig. 4 compares the calculated current from the full TDDFT or independent particle approximation simulations for laser parameters similar to the experiment, but taken at an even higher laser power to try to induce larger correlations. The calculated current in both cases agrees remarkably well, indicating that electronic interactions do not play any role in the main mechanisms observed and discussed in the main text. Finally, the fully coherent simulations show the same behaviour as the experimental set-up, suggesting that electron–phonon scattering does not play a dominant role in this experiment.

We further computed the relative occupations of the different valence and conduction bands after the laser pulse ends by integrating *g*_*n*_(**k**) from equation (11) over *k*-space. From this analysis, we find that, in typical experimental conditions, over 99.999% of the excitations occur in the first valence and first conduction bands. This result, together with the absence of dynamical interactions shown in Supplementary Fig. 4, validates the model two-band tight-binding Hamiltonian used in the analytic approach^47^.

## Online content

Any methods, additional references, Nature Portfolio reporting summaries, source data, extended data, supplementary information, acknowledgements, peer review information; details of author contributions and competing interests; and statements of data and code availability are available at https://doi.org/10.1038/s41567-026-03429-7.

## Supplementary information

Supplementary Information Supplementary Figs. 1–9 and Text.

## Source data

Source Data Figs. 2 and 4 Data for Figs. 2 and 4.

Source Data Fig. 3 Data for Fig. 3.

## Data Availability

Source data are provided with this paper. All other data that support the plots within this article and other findings of this study are available from the corresponding authors upon reasonable request. These datasets comprise raw multiparameter scans (phase, polarization and field-strength dependent photocurrent traces) together with associated calibration and TDDFT simulation outputs, whose structure, coordinate conventions and processing pipeline require substantial context to interpret; we are happy to work directly with interested readers to provide the relevant subsets and analysis code needed for their specific use case.

## Code availability

The Floquet, time-dependent Schrödinger equation and photocurrent-analysis code used to generate the results of this study are available from the corresponding authors upon reasonable request. Octopus code used for time-dependent density functional theory simulations is available open access^55^.
