# Supplementary material for: Optical control of electrons in a Floquet topological insulator
Source: Nat Phys. 2026 Aug 31;22(10):1607–14. doi: 10.1038/s41567-026-03429-7 (PMC13658027; doi:10.1038/s41567-026-03429-7)
Supplement: Supplementary file 1 [file 41567_2026_3429_MOESM1_ESM.pdf]

nature physics

Supplementary information

https://doi.org/10.1038/s41567-026-03429-7

# Optical control of electrons in a Floquet topological insulator

In the format provided by the authors and unedited

**Optical control of electrons in a Floquet topological insulator**

**Supplementary Information**

## Contents

Supplementary Text ..... S2

Comparison to prior coherent-control work at 800 nm ..... S2

Comprehensive symmetry analysis ..... S2

Emergent Chern numbers ..... S4

ARPES Simulation ..... S4

Pulse Characterization ..... S5

Fig. S1: Phase stability of the collinear interferometer ..... S5

Fig. S2: Floquet topological insulating state in graphene ..... S6

Fig. S3: Large bandgap Floquet topological insulating states in graphene ..... S7

Fig. S4: Comparison of calculated current expectation value ..... S7

Fig. S5: Sub-cycle dynamical symmetry selection rules ..... S8

Fig. S6: Emerging Chern numbers from two-color driving ..... S9

Fig. S7: Comparison of FTI states ..... S10

Fig. S8: Calculated ARPES spectra of dressed graphene ..... S11

Fig. S9: Optical Pulse Characterization ..... S11

**Supplementary Text**

*Comparison to prior coherent-control work at 800 nm*

Our own previous work on coherent control in graphene [1] used a much smaller $E_{2\omega}$:$E_{\omega}$ ratio ($\sim$ 3.5%) at 800 nm, compared to 75% at 1550 nm here. Floquet physics predicts a strong dependence on the pump frequency: the fundamental gap scales as $1/\omega^3$ [2], so a factor of two change in pump frequency changes the fundamental gap by a factor of eight. This is consistent with the qualitatively different power-law behavior observed in the present work compared to the prior 800 nm measurements.

*Comprehensive symmetry analysis*

To investigate the origin of the photocurrents, we model the time-dependent dynamics with both analytic and *ab-initio* methods. In figure S5 we present the CNR analysis discussed around Figure 5 of the main text again, highlighting the local symmetries, for clarity. Figures S5a–l show the residual conduction band distributions for the COR, CNR, and TD waveforms obtained from TDDFT. Analyzing all six K and K' points allows us to look at the dynamical selection rules for the respective waveforms globally, while the zoom-ins (figs. S5g–l) highlight the intra-valley symmetries.

We analyze the dynamical selection rules by the residual conduction band populations for two orthogonal phases of both the COR, CNR, and TD waveforms. After excitation with the COR waveform (figs. S5a–b) we can see one mirror axis present in each of the graphene band structures corresponding to the waveform symmetry. For $\varphi_{\omega-2\omega}= \pi/2$ (fig. S5a), we observe a left/right symmetry in the conduction band population, resulting in no current generation, with a dominate symmetry axis through M–Γ–M', addressing the K and K' points equivalently, rendering $\rho^c$ K and K' symmetric. The zoom-in (fig. S5g) highlights the crescent features showing this vertical mirror axis between the K and K' points. When $\varphi_{\omega-2\omega}= 0$ (figs. S5b, h), our waveform's principle symmetry axis changes to the K–Γ–K' axis. This disconnects the K and K' points and allows intra-valley momentum imbalances and a net current.

For the excitation with a CNR waveform (figs. S5c , d), similar arguments can be made, now with the waveforms exhibiting two additional minor mirror axes (dashed orange lines) corresponding to the three-fold rotational symmetry of the waveform. Importantly, the time reversal symmetry (purple color bar) is coupled to the primary mirror axis (green). Critically, shown in the $\varphi_{\omega-2\omega}=$

$\pi/2$ waveform, the symmetry axis connects K and K' (a M-Γ-M' axis), resulting in the inability to generate a valley polarization. With $\varphi_{\omega-2\omega}$= 0, the waveform's symmetry axis (K-Γ-K') now disconnects the K and K' points, resulting in possible valley polarization. The zoom-ins of the CNR population (fig. S5i, j) reveal the local symmetries (dashed orange lines) within the K and K' valleys.

After excitation with the TD waveform (figs. S5e, f) we can see one mirror axis present in each of the graphene band structures corresponding to the waveform symmetry. For $\varphi_{\omega-2\omega}$= $\pi/2$ (fig. S5e), we observe a left/right symmetry in the conduction band population, resulting in no current generation, with a dominate symmetry axis through M–Γ–M', addressing the K and K' points equivalently, rendering $\rho^{c}$ K and K' symmetric. The zoom in (fig. S5k) highlights the crescent features showing this vertical mirror axis between the K and K' points. When $\varphi_{\omega-2\omega}$= 0 (figs. S5f, l), our waveform's principle symmetry axis changes to the K–Γ–K' axis. This disconnects the K and K' points and allows intra-valley momentum imbalances and a net current.

To understand the emergence of these characteristic patterns (seen in figs. S5h, g, and l) and the underlying valley control on a sub-cycle time scale, it is insightful to discuss a picture of time-dependent inter- and intraband electron dynamics in the undressed band structure. We highlight these dynamics for three exemplary electrons with initial momenta equidistant to the respective K and K' point (figs. S5h, j, l, colored circles). Figures S5n, p, r, show the respective sub-cycle evolution of the interband dipole coupling $V^{\text{cv}}(t)$ induced by the COR, CNR, and TD waveform, respectively (at $\varphi_{\omega-2\omega}$= 0). The stark inter-valley differences in $|V^{\text{cv}}|$ occurring during the center optical cycle (figs. S5m, o, q; shaded areas) become clear with a fundamental field strength of 1 V/nm applied here for visibility.

The COR waveform drives the right marked electron in the K' valley (fig. S5h) close to the Dirac point, resulting in a large excitation probability (fig. S5n), whereas it is suppressed for the other two marked electrons, which are driven away from the Dirac point. Hence all three exemplary electrons are shown to assist in the generation of intra-valley momentum asymmetry, observed in the crescent shaped distribution of fig. S5h and measured as a large current (Figure 4b of the main text). The three marked electrons in the K valley contribute likewise.

In contrast, the CNR waveform drives all three exemplary electrons in the K' valley (fig. S5j) equally close to the Dirac point. At the K point, the same three electrons come close to the Dirac point only for a highly confined duration at the trefoil peaks, lowering their excitation probability. In the dipole coupling (fig. S5p) this is reflected in a larger amplitude of $|V^{\text{cv}}|$ for each electron around K' compared to K. Importantly, these valley-specific differences in interband coupling result

in a valley-polarized conduction band population. In addition, since the relative amplitudes of the marked points are not exchanged between K and K' (as is the case for the COR waveform, fig. S5n), there is no intra-valley momentum asymmetry. This allows us to identify the drastic reduction of the measured current (Figure 4b of the main text) as an experimental generation of valley-polarized graphene.

The TD waveform drives the right marked electron in the K' valley (fig. S5l) equivalently close to the Dirac point, resulting in an approximately equal excitation probability (fig. S5r). The three exemplary electrons in the K' valley behave similarly to the CNR case. Electrons around the K valley are driven differently. The electron on the mirror plane is driven close to the dirac point, while the other two are not driven to the dirac point (fig. S5r). All three exemplary electrons in the K valley are shown to assist in the generation of intra-valley momentum asymmetry, observed in the crescent shaped distribution of fig. S5l and measured as a large current (Figure 4b of the main text). The intravalley-asymmetry present in only one of the two valleys produces valley current.

*Emergent Chern numbers*

To verify that the two pulses can be split into a constant dressing pulse and a drive pulse, we calculate the emerging Chern numbers for all three combinations of driving fields using the Hamiltonian from the high frequency expansion [3]. Importantly, the Chern number remains identical (at $\pm 1$) regardless of the ellipticity of the $2\omega$ field. The Berry curvature is calculated identically to the Methods section [4]. We present the three cases in figure S6 and highlight that the topological state is equivalent for all three cases.

Further, for the specific case of the anomalous Hall current, we compare the Floquet band structure of only the dressing ($\omega$) pulse with the dressing and drive ($\omega + 2\omega$) pulses combined (fig. S7). We see that the Berry curvature is largely determined by the dressing ($\omega$) pulse while the driving ($2\omega$) pulse has less impact on the Berry curvature due to the weaker vector potential and higher frequency. This is in line with the results from the high frequency expansion shown above.

*ARPES Simulation*

We calculate the time and angle resolved photoelectron spectrum (Tr-ARPES) of graphene (fig. S8) under identical light dressing conditions to the experiment, with conduction band doping. The results are calculated ab-initio with state-of-the-art simulations, and including doping and

electron-electron interactions within TDDFT (with the same approach employed in ref. [5]). We see gap sizes and Floquet bands that match those predicted by the model Hamiltonain discussed earlier, clearly demonstrating in this regime, with near-infrared driving presented in this paper, an FTI state is created with a topological gap that should be measurable.

*Pulse Characterization*

We characterize the pulses after the BiBO crystal by second harmonic generation frequency resolved optical gating (SHG-FROG). We obtain a 213 fs pulse for the fundamental and a 110 fs pulse for the second harmonic, as shown in figure S9.

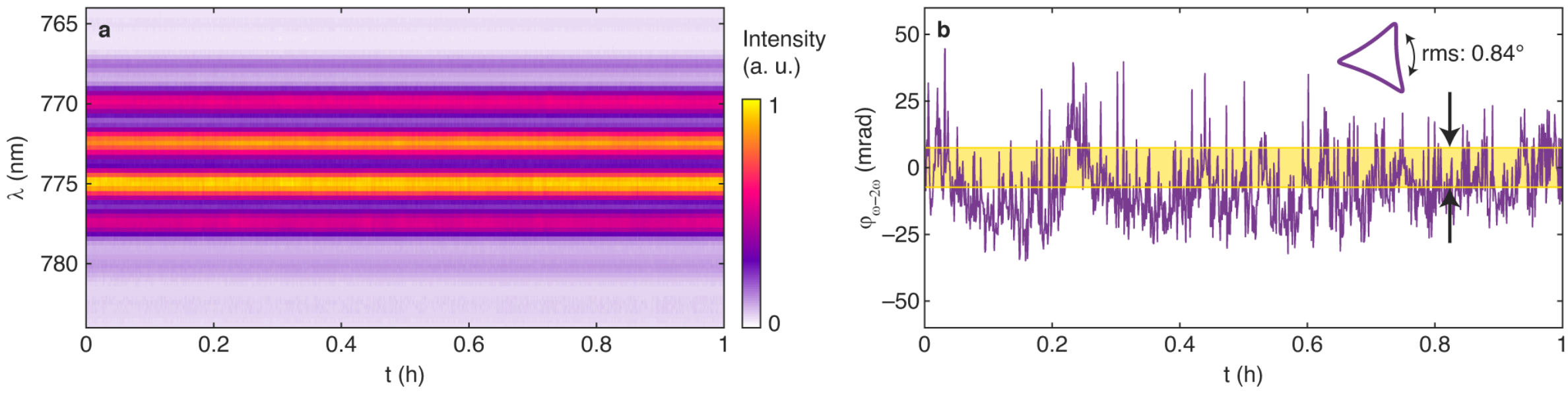


FIG. S1. **Phase stability of the collinear interferometer.** **a**, Interference fringes at $2\omega$ measured every 35 ms for 1 hour with a spectrometer integration time of 10 ms. **b**, Reconstructed jitter of $\varphi_{\omega-2\omega}$ obtained from a cosine square fit, yielding a root mean square of 14.6 mrad (0.84°) for the angular stability of the bichromatic Lissajous figures (yellow shading).

[1] Heide, C. et al. Electronic coherence and coherent dephasing in the optical control of electrons in graphene. Nano Letters **21**, 9403–9409 (2021).

[2] Rudner, M. S. & Lindner, N. H. Band structure engineering and non-equilibrium dynamics in Floquet topological insulators. Nature Reviews Physics **2**, 229–244 (2020).

[3] Eckardt, A. & Anisimovas, E. High-frequency approximation for periodically driven quantum systems from a Floquet-space perspective. New Journal of Physics **17**, 093039 (2015).

[4] Xiao, D., Chang, M.-C. & Niu, Q. Berry phase effects on electronic properties. Reviews of Modern Physics **82**, 1959–2007 (2010).

[5] Neufeld, O., Hübener, H., Giovannini, U. D. & Rubio, A. Tracking electron motion within and outside of Floquet bands from attosecond pulse trains in time-resolved ARPES. Journal of Physics: Condensed Matter **36**, 225401 (2024).

[6] Oka, T. & Aoki, H. Photovoltaic Hall effect in graphene. Physical Review B **79**, 081406(R) (2009).

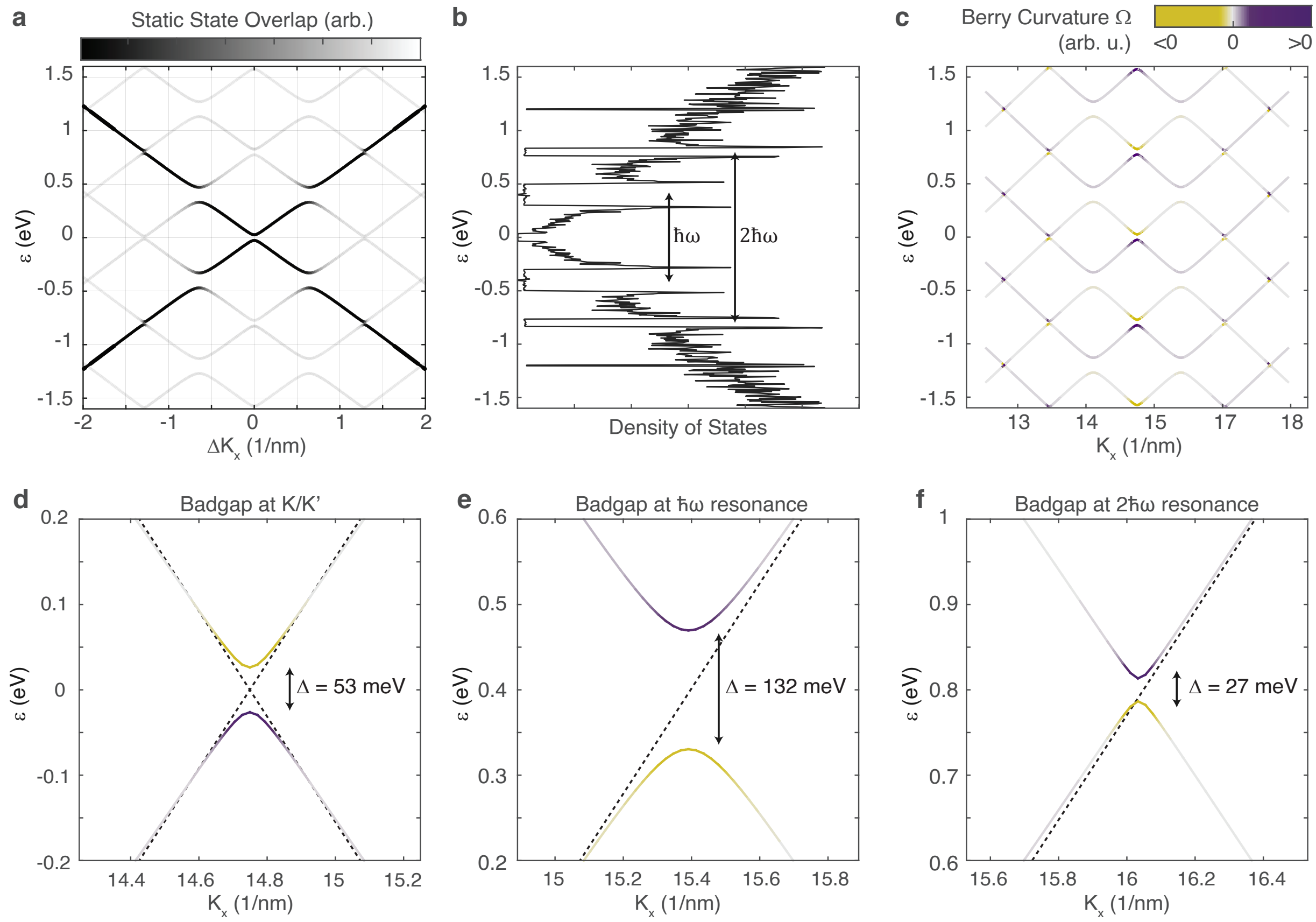


FIG. S2. **Floquet topological insulating state in graphene. a** Calculated Floquet band structure for graphene under experimental illumination conditions, static state overlap calculated from [6, 7]. **b** Density of states for the dressed band structure in (a) showing bandgaps at the Dirac point, one, and two-photon resonances. **c** Berry curvature of the dressed band structure[4]. **d–f** Zoom in of the topological band gaps and Berry curvature at the K point (**d**), $\hbar\omega$ resonance (**e**, 10x Berry curvature), and two photon resonance (**f**, 10x Berry curvature).


[7] Sato, S. A. et al. Microscopic theory for the light-induced anomalous Hall effect in graphene. Physical Review B **99**, 214302 (2019).

[8] Lesko, D. M. B. et al. A six-octave optical frequency comb from a scalable few-cycle erbium fibre laser. Nature Photonics **15**, 281–286 (2021).

[9] Lesko, D. M. B., Chang, K. F. & Diddams, S. A. High-sensitivity frequency comb carrier-envelope-phase metrology in solid state high harmonic generation. Optica **9**, 1156 (2022).

[10] Weitz, T., Heide, C. & Hommelhoff, P. Strong-field Bloch electron interferometry for band-structure retrieval. Physical Review Letters **132**, 206901 (2024).

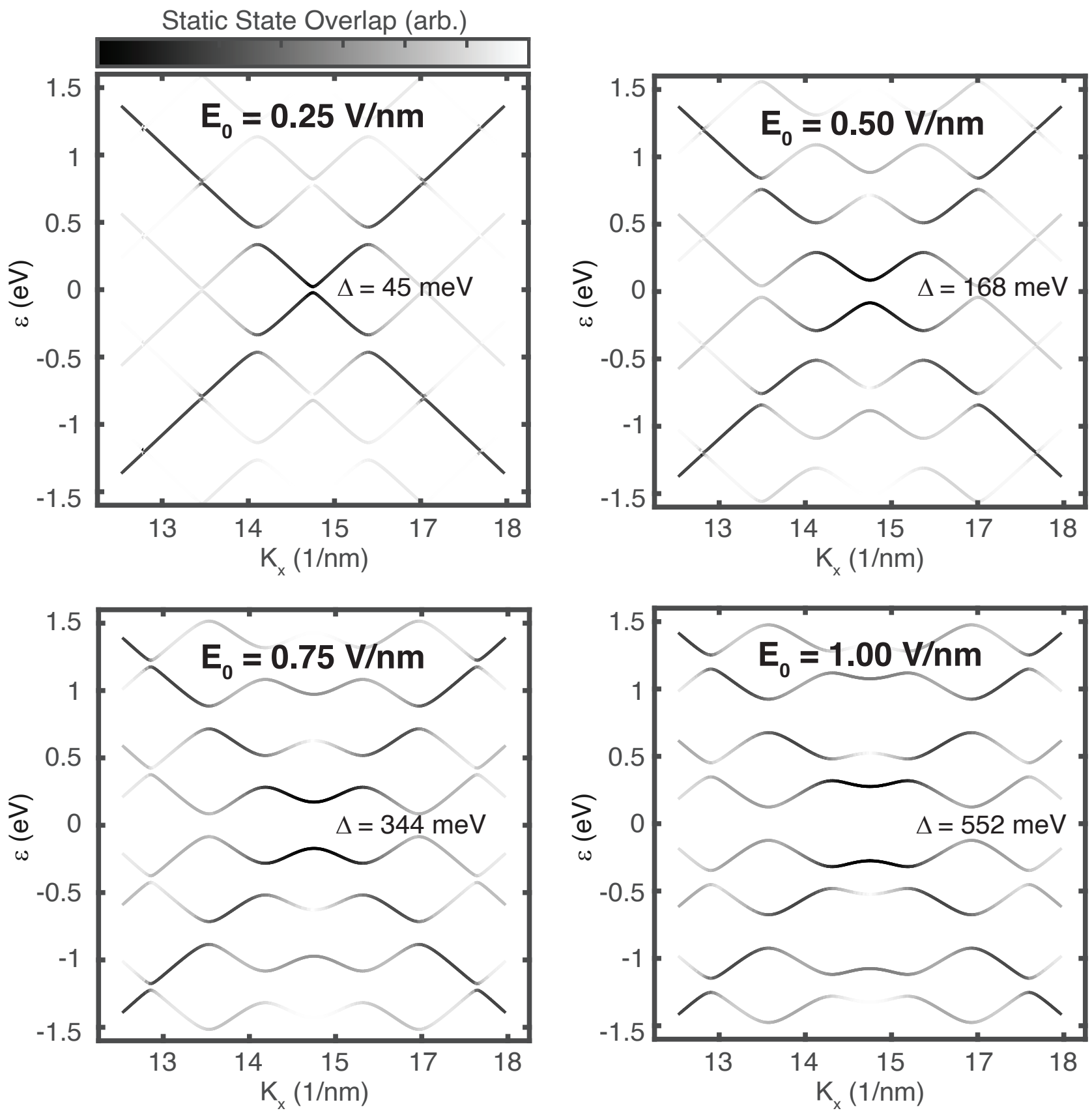


FIG. S3. **Large bandgap Floquet topological insulating states in graphene.** Calculated Floquet band structure for graphene under a variety of experimentally feasible field strengths at 1550 nm [8, 9], static state overlap calculated from [6, 7].

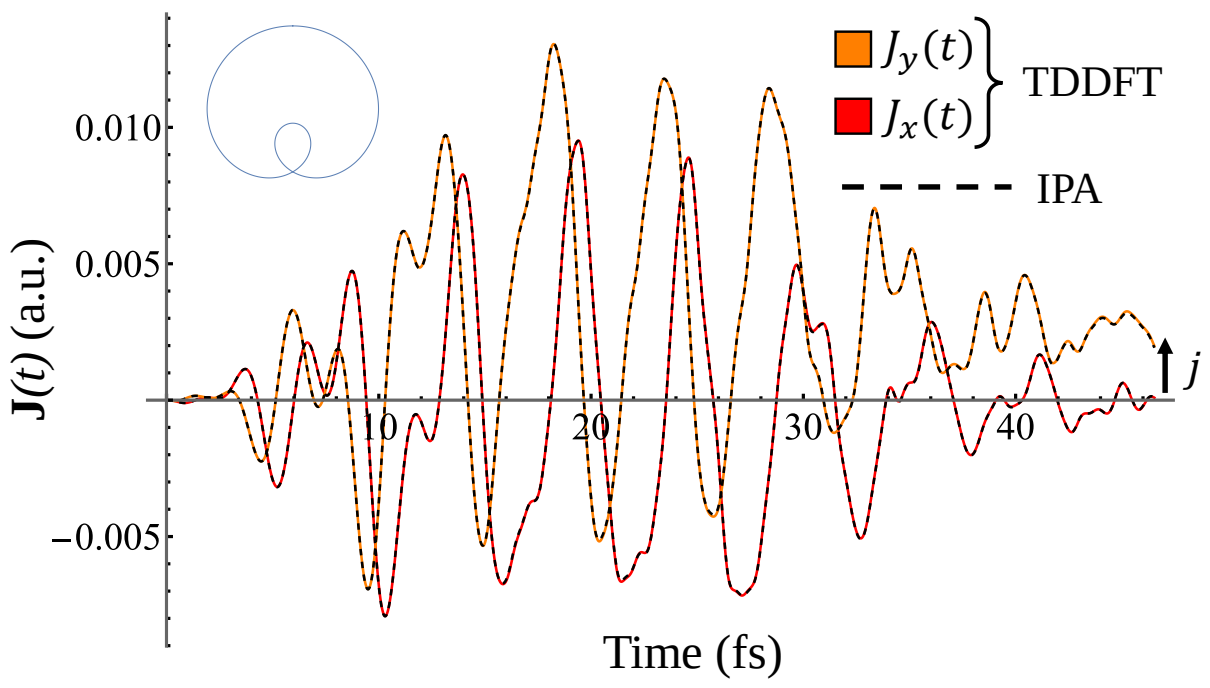


FIG. S4. **Comparison of calculated current expectation value**, $\mathbf{J}(t)$ from Eq. 10 of the main text, with a full TDDFT calculation, or within the IPA with frozen electronic interactions. The system's response is almost identical in both cases, indicating the interactions are negligible. Calculated for co-circular case at a high laser power of $10^{11}$ W/cm$^2$ with 1550 nm driving.

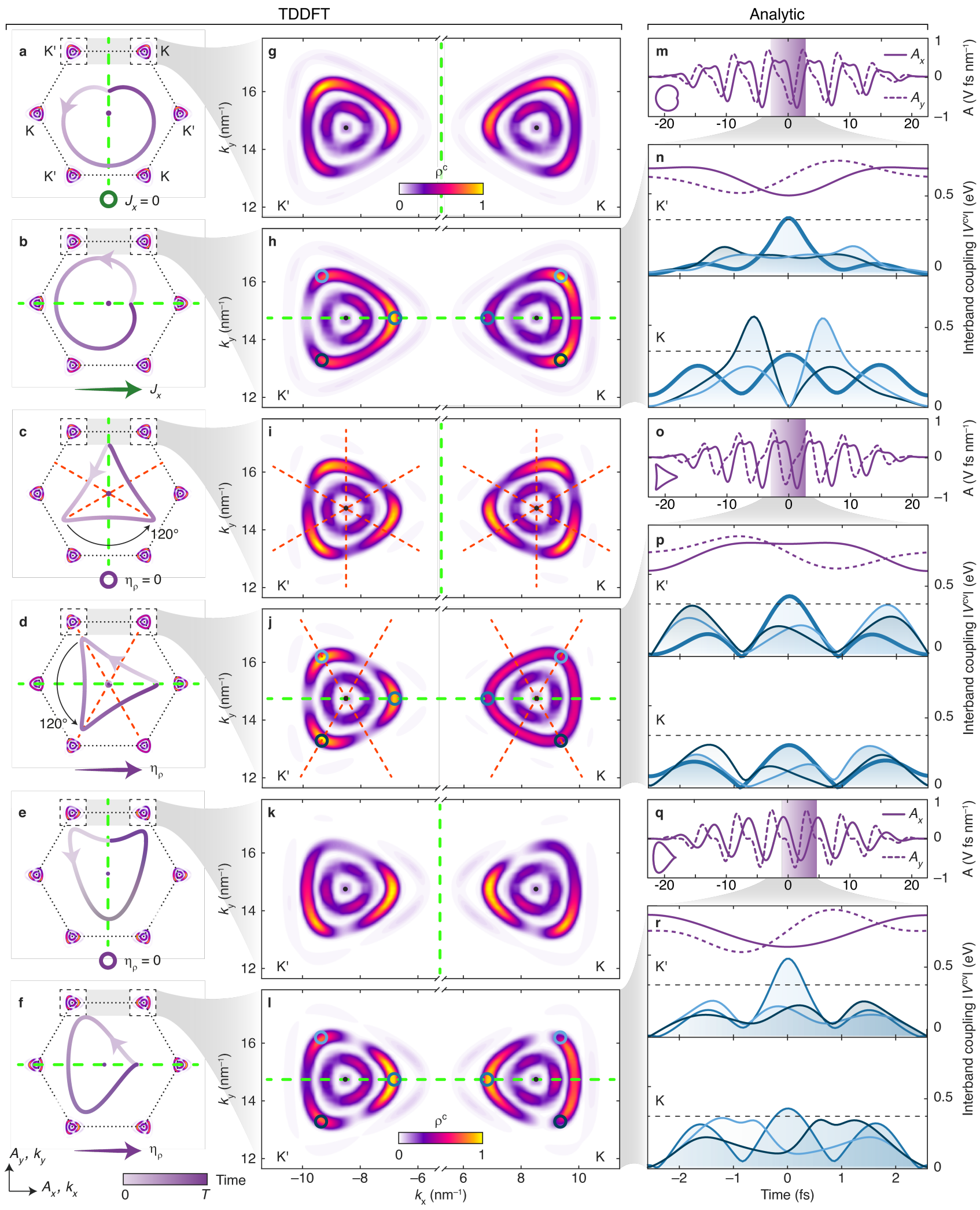


FIG. S5. **Sub-cycle dynamical symmetry selection rules. a–d**, Temporal shape and symmetries of the vector potential Lissajous figures (purple lines) and the resulting symmetries of residual momentum distributions $\rho^{c}$ imprinted to the first Brillouin zone (dotted hexagons) for the COR (**a, b**), CNR (**c, d**), and TD (**e, f**) waveform for $\varphi_{\omega-2\omega} = \pi/2$ and 0 respectively. The momentum distributions are computed with TDDFT (see Methods for details). Green (orange) dashed lines indicate global (valley-specific) symmetry axes, determined by both waveform and time periodicity (see purple color bar). **g, l**, Close-ups of $\rho^{c}$ around K and K' as indicated in **b**, **d**, and **f**. For all cases, an eight-cycle pulse (super-sine envelope, see vector potential in **m**, **o**, and **q**) is applied with 1550 nm fundamental wavelength with a peak field strength $E_0 = 0.27$ V/nm and 75 % $2\omega$ field admixture. **m**, **o**, **q**, COR (**m**), CNR (**o**), and TD (**q**) vector potential waveform for $\varphi_{\omega-2\omega} = 0$. The shaded areas indicate the center optical cycle respectively. **n**, **p**, **r**, Temporal profile of the interband dipole coupling $|V^{\mathrm{cv}}|$ [10] induced by the waveforms shown in **m**, **o**, and **q**, respectively. The different shades of blue refer to initial momenta in the K and K' valley as marked with circles in panels **h–l**. The dashed black guide lines compare the $|V^{\mathrm{cv}}|$ amplitudes at K and K'.

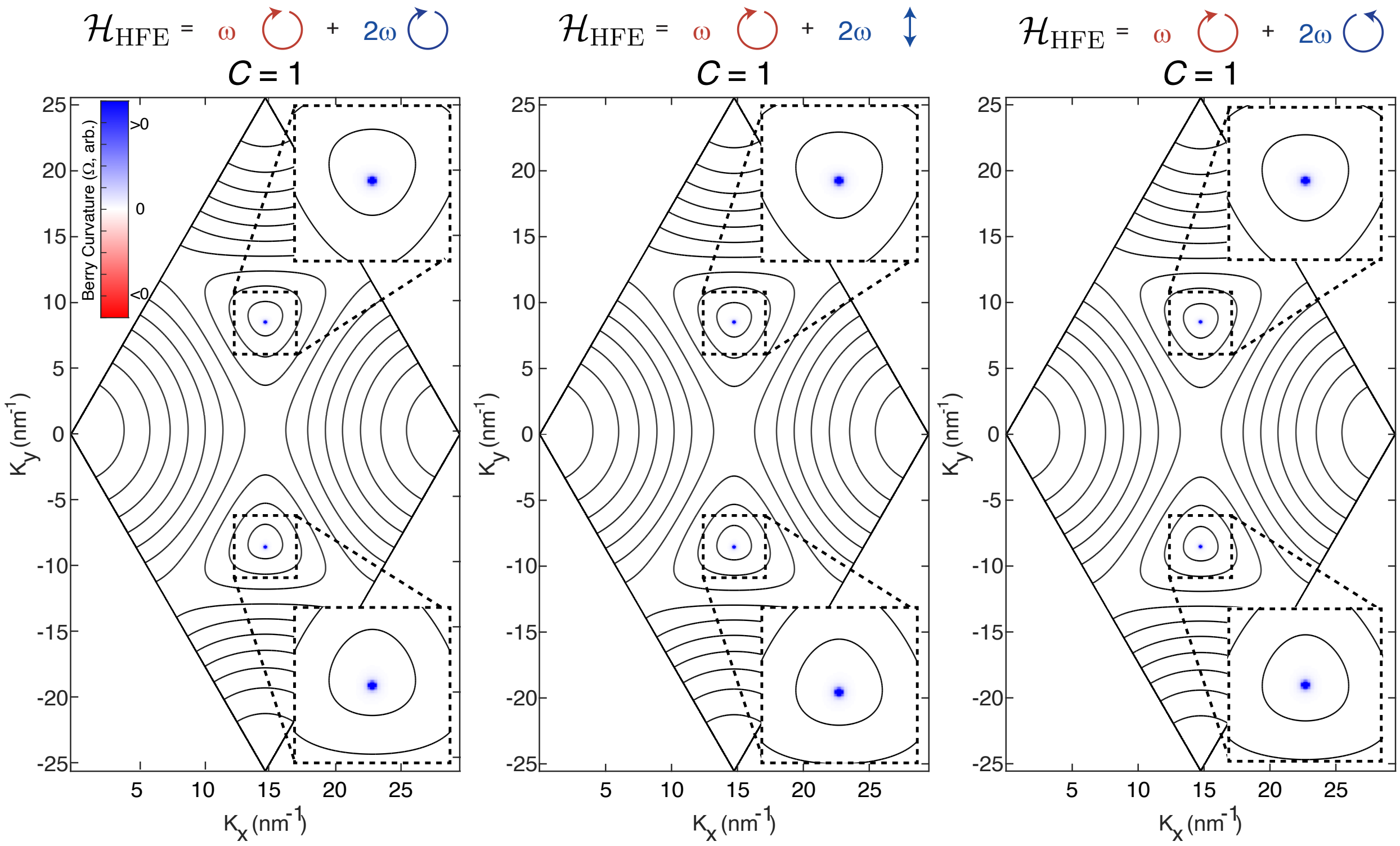


FIG. S6. **Emerging Chern numbers from two-color driving.** We plot the Berry curvature of the full Brillouin zone for each of the two color driving cases using the high frequency expansion. Color coding for the Berry curvature, with the black solid lines representing equipotential surfaces for excitation with the fundamental field. The Chern number is equivalent for all three of the cases.

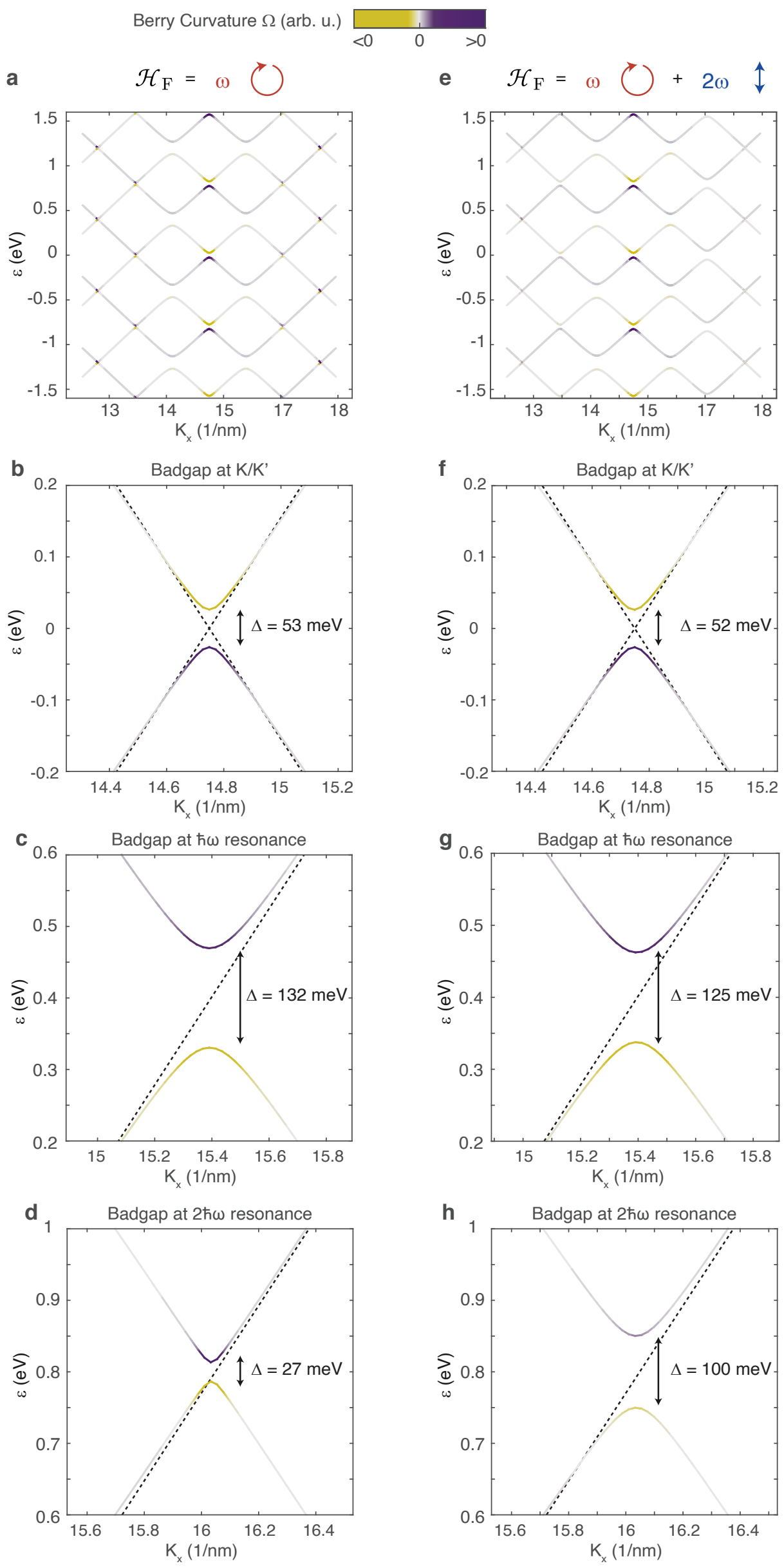


FIG. S7. **Comparison of FTI states.** We plot the Floquet band structure and Berry curvature for $\omega$ only (**a–d**) as well as $\omega + 2\omega$ (**e–h**).

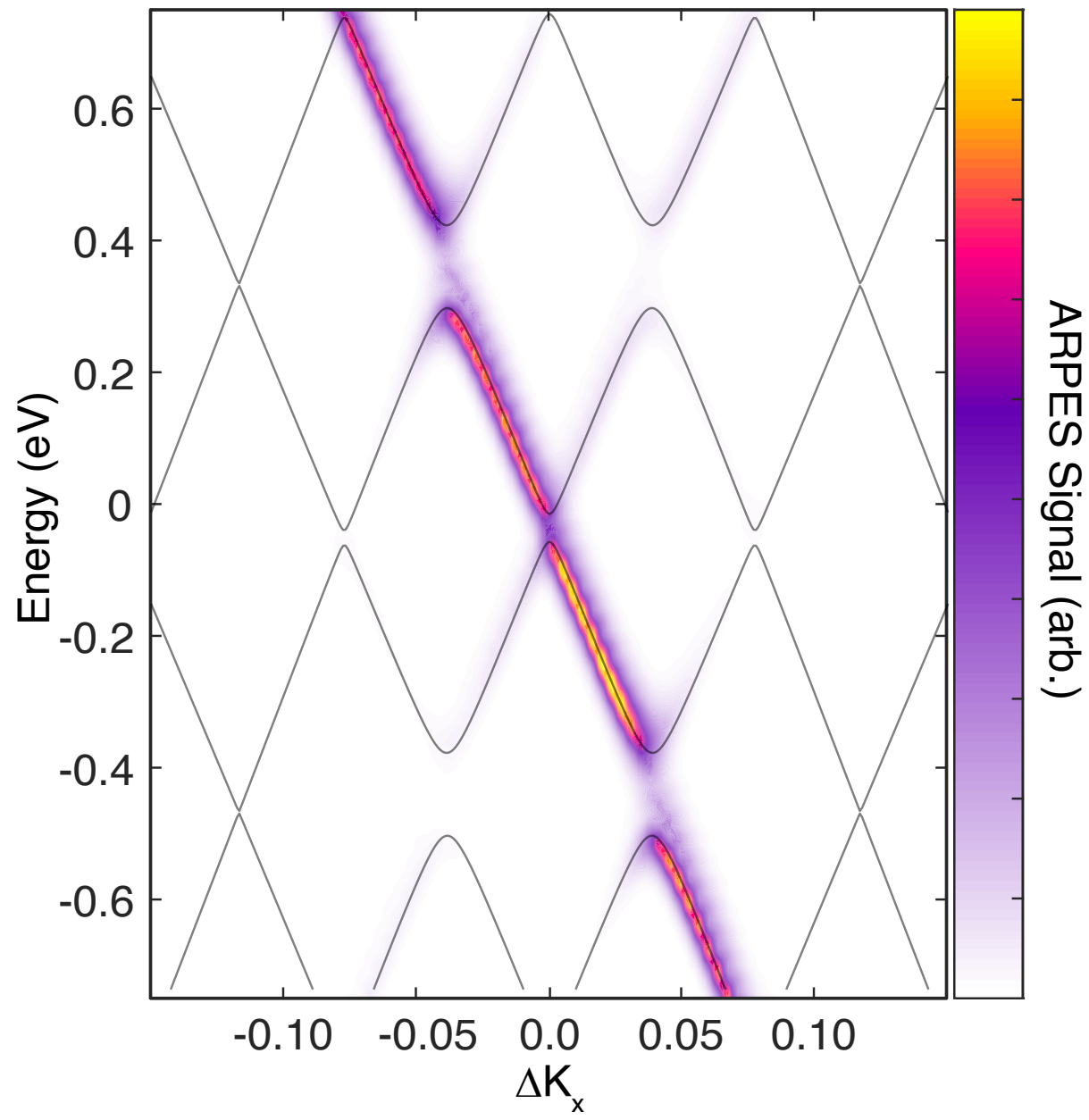


FIG. S8. **Calculated ARPES spectra of dressed graphene.** ARPES spectra of n-doped graphene dressed with an optical field identical to the one in this manuscript showing an evident bandgap at K and the first avoided crossing.

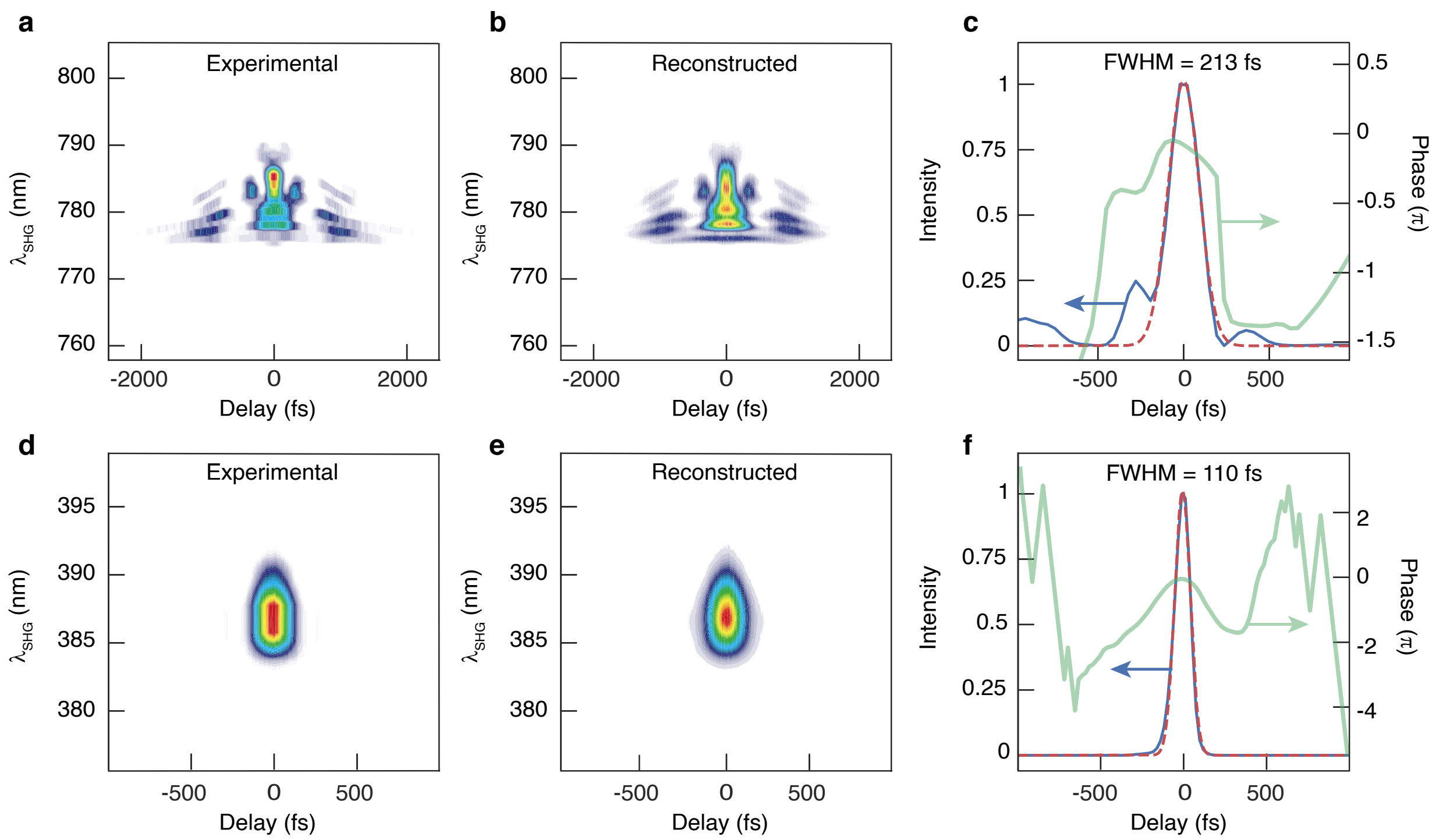


FIG. S9. **Optical Pulse Characterization** We measure the SHG-FROG spectrogram for $\omega$ (**a**) and reconstruct (**b**) a 213 fs pulse ((**c**)). The same is done for the $2\omega$ pulse (**d**–**f**) revealing a 110 fs pulse.
